# Scoping review of the effectiveness of 10 high-impact initiatives (HIIs) for recovering urgent and emergency care services

**DOI:** 10.1136/bmjoq-2024-002906

**Published:** 2024-09-18

**Authors:** Christopher Carroll, Burak Kundakci, Amber Muhinyi, Anastasios Bastounis, Katherine Jones, Anthea Sutton, Steve Goodacre, Carl Marincowitz, Andrew Booth

**Affiliations:** 1 School of Medicine and Population Health, University of Sheffield, Sheffield, UK

**Keywords:** Emergency department, Ambulatory care, Health policy, Health services research, Prehospital care

## Abstract

**Introduction:**

Prolonged ambulance response times and unacceptable emergency department (ED) wait times are significant challenges in urgent and emergency care systems associated with patient harm. This scoping review aimed to evaluate the evidence base for 10 urgent and emergency care high-impact initiatives identified by the National Health Service (NHS) England.

**Methods:**

A two-stage approach was employed. First, a comprehensive search for reviews (2018–2023) was conducted across PubMed, Epistemonikos and Google Scholar. Additionally, full-text searches using Google Scholar were performed for studies related to the key outcomes. In the absence of sufficient review-level evidence, relevant available primary research studies were identified through targeted MEDLINE and HMIC searches. Relevant reviews and studies were mapped to the 10 high-impact initiatives. Reviewers worked in pairs or singly to identify studies, extract, tabulate and summarise data.

**Results:**

The search yielded 20 771 citations, with 48 reviews meeting the inclusion criteria across 10 sections. In the absence of substantive review-level evidence for the key outcomes, primary research studies were also sought for seven of the 10 initiatives. Evidence for interventions improving ambulance response times was generally scarce. ED wait times were commonly studied using ED length of stay, with some evidence that same day emergency care, acute frailty units, care transfer hubs and some in-patient flow interventions might reduce direct and indirect measures of wait times. Proximal evidence existed for initiatives such as urgent community response, virtual hospitals/hospital at home and inpatient flow interventions (involving flow coordinators), which did not typically evaluate the NHS England outcomes of interest.

**Conclusions:**

Effective interventions were often only identifiable as components within the NHS England 10 high-impact initiative groupings. The evidence base remains limited, with substantial heterogeneity in urgent and emergency care initiatives, metrics and reporting across different studies and settings. Future research should focus on well-defined interventions while remaining sensitive to local context.

**WHAT IS ALREADY KNOWN ON THIS TOPIC**

- 10 initiatives were highlighted by National Health Service England as having a potential ‘high impact’ on urgent and emergency care performance. However, no formal review of the published evidence on these initiatives and their impact on emergency department (ED) wait times or ambulance response times had previously been undertaken.

**WHAT THIS STUDY ADDS**

- Some evidence suggests that some ‘high-impact initiatives’, such as same day emergency care, acute frailty units, care transfer hubs and some in-patient flow interventions might reduce ED wait times; there is weak evidence for the beneficial effect of urgent community response, prehospital telemedicine and some in-patient flow interventions on some time-based outcomes for ambulances. However, the evidence base overall is limited in both quantity and quality.

**HOW THIS STUDY MIGHT AFFECT RESEARCH, PRACTICE OR POLICY**

- Future research and policy should evaluate well-defined and specific interventions thereby allowing an enhanced focus on local variability and variation.

## Introduction

 Prolonged ambulance response times and excessive emergency department (ED) wait times are significant challenges faced by urgent and emergency care (UEC) systems worldwide. Delays in accessing emergency care can negatively impact patient outcomes and healthcare system efficiency. In the UK, the Royal College of Emergency Medicine estimates there may up to 300 deaths a week associated with ED delays.^1^ Recent UK research has demonstrated that delays to hospital inpatient admission for patients in excess of 5 hours from time of arrival at the ED are associated with an increase in all-cause 30-day mortality.^2^ Beyond 5 and up to 12 hours, delays cause a predictable dose–response effect; for every 82 admitted patients whose time to inpatient bed transfer is delayed beyond 6–8 hours, there is one extra death.^2^ A Swedish study has further demonstrated that survival to 30 days after a witnessed out-of-hospital cardiac arrest decreases as ambulance response times increase.^3^

In January 2023, National Health Service (NHS) England published a ‘Recovery Plan’ that set out an ambition for a health system that provides more and better care in people’s homes, gets ambulances to people more quickly when they need them, sees people faster when they go to hospital and helps people safely leave hospital having received the care they need.^4^ While stating that improvements were required across patient pathways in acute, community and mental health settings generally, the plan specified two ambitions in the short term (to the end of 2023/2024) in relation to UEC provision: (1) a 30 min mean response time for category 2 ambulance and (2) 76% performance in ED wait times, measured through the 4-hour target.^4^

To support the recovery plan, NHS England and the UK National Institute of Health Research have commissioned both primary research and evidence syntheses to evaluate existing initiatives. As part of this wider evaluation process, the authors were commissioned to conduct scoping reviews relating to the 10 high-impact initiatives identified by NHS England as having the potential to deliver on the commitments set out in the Recovery Plan. Some of these initiatives, such as same day emergency care (SDEC) and acute frailty services, were mandated by the NHS England Long Term Plan in 2019.^5^ These 10 initiatives are listed and briefly outlined in table 1 (fuller details are provided in online supplemental appendix 1). The rationale for these initiatives was to reduce pressure on hospital bed occupancy by managing or diverting appropriate patients elsewhere, expediting discharge and reducing admission rates from the ED.^6 7^ This might be described as a focus on inputs and outputs within the system, rather than interventions or models seeking to affect the ‘throughput’ or ‘flow’ of patients.^8 9^ The review was conducted between January and March 2024.

**Table 1:** The 10 high-impact Initiatives

**Urgentcommunity response** increasing volume and consistency of referrals to improve patient care and ease pressure on ambulance services and avoid admission (NHS England 2022).	**Community beds** reducing variation in inpatient care and length of stay, including mental health, by implementing in-hospital efficiencies and bringing forward discharge processes.
**Samedayemergencycare(SDEC)** reducing variation in SDEC provision by providing guidance about operating a variety of SDEC services for at least 12 hours per day, 7 days per week (NHS England 2021).	**Intermediate care** supporting the operationalisation of ongoing demand and capacity planning, including through improved use of data to improve access to and quality of intermediate care including community rehab.
**Acute frailty** reducing variation in acute frailty service provision. Improving recognition of cases that could benefit from specific frailty services and ensuring referrals to avoid admission (NHS England 2019)	**Singlepoint ofaccess** driving standardisation of urgent integrated care co-ordination which will facilitate whole system management of patients into the right care setting, with the right clinician or team, at the right time. This should include mental health crisis pathways and alternatives to admission, for example, home treatment
**In-patient flow** reducing variation in inpatient care (including mental health) and length of stay for key iUEC pathways/conditions/cohorts by implementing in-hospital efficiencies and bringing forward discharge processes for pathway 0 patients.	**Acuterespiratoryinfectionhubs (ARI hubs)** support consistent roll-out of services, prioritising ARI, to provide same day urgent assessment with the benefit of releasing capacity in ED and general practice to support system pressures.
**Caretransferhubs** implementing a standard operating procedure and minimum standards for care transfer hubs to reduce variation and maximise access to community rehabilitation and prevent re-admission to a hospital bed.	**Virtual wards** standardising and improving care across all virtual ward services to improve the level of care to prevent admission to hospital and help with discharge

ED, emergency department; iUEC, integrated urgent emergency care; NHS, National Health Service

### Objectives

This scoping review aimed to summarise the emerging, published evidence base for the impact of the NHS England 10 high-impact initiatives on the UEC performance metrics of ED wait times and ambulance response times (as specified in the NHS England ‘Recovery Plan’2023.^4^

## Methods

Given the size of the evidence base and the need to deliver a timely and up-to-date review that would assist policy-makers, the team conducted a two-stage rapid scoping review of the evidence. The first stage involved identifying relevant review-level evidence on the high-impact initiatives and related interventions (tier 1 evidence); and, in the event that this level of evidence was not considered substantive, a second stage involved identifying relevant primary research (tier 2 evidence, eg, randomised controlled trials (RCTs), quasi-experimental studies and observational studies). The Preferred Reporting Items for Systematic Reviews and Meta-Analyses (PRISMA) extension for Scoping Reviews checklist was used for this review^10^ and can be found insupplemental file(online supplemental appendix 2). A protocol was registered with PROSPERO (CRD: 42024502585).

### Search strategy and eligibility criteria

Retrieval of evidence followed our two-stage approach. First, we conducted a comprehensive search of three electronic databases or resources (MEDLINE, Epistemonikos and Google Scholar) for systematic reviews and reviews applying systematic principles, combined with terms for the initiatives and terms for UEC settings (tier 1 evidence). This search retrieved all reviews that had been conducted in a UEC setting by limiting to reviews (MEDLINE, Epistemonikos) or using review terms (Google Scholar) and was conducted in December 2023. Given the potential heterogeneity of interventions and their description and the review focus on outcomes, the review team also conducted an ‘outcomes’ search consisting of complementary full-text searching of Google Scholar for reviews with references to (1) overcrowding, (2) ED wait(ing) times and (3) ambulance response. This broad ‘outcome’-level search was conducted to ensure that potentially relevant items that might have failed to specify a relevant initiative in title or abstract could be retrieved and checked. When review-level evidence was found to be scarce, experienced information specialists conducted supplementary searches for tier 2 evidence combining terms for the initiatives with UEC settings terms in a minimum of two databases (MEDLINE, HMIC). Tier 2 searches were conducted in February 2024.

The date limits for all searches were from January 2018 to December 2023 because this period both marginally predates the NHS Long Term Plan (2019) and extends to the Plan’s 2023–2024 horizon. Primary publications were restricted to English only, although non-English sources could be covered within individual systematic reviews. Search strategies are provided in online supplemental appendix 3.

Studies were included if they were a review (tier 1) or primary research study (tier 2) reporting: (1) one of the 10 high-impact initiatives, or potentially similar or relevant interventions and (2) at least one of the two ‘headline metrics’ as identified by NHS England for UEC, namely ambulance response times or ED wait times (including ED length of stay (LOS)), or other ambulance-centric or time-sensitive ED metrics. The full eligibility criteria are outlined in table 2.

**Table 2:** Eligibility criteria

Setting	Urgent and emergency care (UEC)Hospital emergency departments (EDs) or settings that offer alternative venues for the delivery of UEC delivery. Studies dealing exclusively with obstetric UEC will be excluded because this is handled separate from the standard UEC system
Population	Adults (over 18 years) and older adults (over 70).Specifically paediatric acute respiratory infection hubs and paediatric single point of access will be included.
High-impact initiativessynonyms and related intervention terms*	1. Urgent community responseRapid response service (RRS); hospice RRS; community-based urgent care service; urgent home care; out-of-hours care
	2. Same day emergency care (SDEC)SDEC; ambulatory emergency care
	3. Acute frailtyFrailty assessment unit; frailty assessment and intervention; frailty-in-urgent-care-settings; older people’s assessment liaison and acute frailty unit/service same day acute frailty services
	4. In-patient flowInterventions to reduce ED exit block (eg, full capacity protocols, escalation protocols—alongside discharge planning and coordination which is the main way of improving in-patient flow. To include greater staff and patient involvement and use of estimated discharge dates). To exclude ‘boarding’
	5. Care transfer hubsDischarge coordination teams; transitional care teams; virtual discharge teams; vommunity discharge teams; transfer of care hub; discharge hub/team/cell/; integrated discharge hub/team/service; single point of access; home first hub/centre; coordination hub/centre; home safe; multi-agency hub; transfer care bureau; care point; community assessment team; discharge command centre; front door and hospital discharge; health and social care hub; intermediate care assessment team; onward care team; right care
	6. Community bedsStep-down beds; transitional care beds; intermediate care beds; rehabilitation beds; community recovery beds to include local authority and NHS maintained beds; P2 beds, D2A beds. Other terms from community beds audit (June 2023)
	7. Intermediate careCommunity-based intermediate care. To include step down, bedded and non-bedded.
	8. Single point of accessUnified access point; integrated urgent care access; front-door access; centralised access to care; single entry point
	9. Acute respiratory infection hubs (ARI hubs)Respiratory infection hubs; respiratory care clinics; respiratory assessment centres/centres; upper respiratory infection clinics; ARI centres/centres; paediatric ARI Hubs
	10. Virtual wardsHospital at home; home-based care; remote patient monitoring; telehealth care; domiciliary care; virtual ward
Outcomes	Time-based outcomes related to the NHS Recovery Plan (2023) in the UEC setting: ED wait times (4 hours target), ED-LOS Ambulance response times (30 min average), including the following metrics, for example, onset to door, time to treatment, ambulance travel times
Study designs	Tier 1: Literature reviews, including systematic reviews, scoping reviews, umbrella reviewsTier 2: Randomised trials, comparative observational studies (including cohort, case–control and before-and-after studies)
Setting	Organisation for Economic Cooperation and Development countries

* Identified with support from NHSE and the Sheffield Centre for Urgent and emergency Care Research () team.

EDemergency departmentLOSlength of stayNHSNational Health ServiceNHSENHS England

### Study selection and data extraction/synthesis

Depending on the size of the evidence base (for tier 1), screening of titles/abstracts and full texts was conducted either by a pair of reviewers (led by a primary reviewer, with decisions checked by a secondary reviewer) or by a single reviewer alone, depending on the size of the evidence base for each initiative. For the large, complementary ‘outcomes’ search, a pair of reviewers started by independently double-screening a 20% sample, to ensure agreement and clarity in the application of eligibility criteria, before dividing the remaining items between them. Full text items were double-screened. Potentially relevant items were then categorised according to one or more of the initiatives and assessed for inclusion by the primary reviewer for each review. Data extraction and synthesis were conducted either by a pair of reviewers (led by a primary reviewer, with decisions and data checked by a secondary reviewer) or by a single reviewer alone. Similar processes were followed for the tier 2 evidence, where applicable. Searches were supplemented by cross-referral between reviewers for the 10 high-impact initiatives.

Relevant items, for tier 1 or tier 2, were summarised directly into evidence tables. Extraction of review-level data (systematic reviews, scoping reviews, etc) included data on first author, year of publication, countries and sample size (n=studies), study design, intervention, population and findings. Data items for primary research study data (quantitative studies) comprised first author, year, country and sample size (n=patients), study design, intervention, population and findings. Across both tiers, outcomes of interest were ambulance response times, other ambulance-related metrics, ED-LOS/wait times, other ED-related time-sensitive metrics.

The evidence base and its findings are reported as a narrative synthesis. The features of target populations, interventions, their implementation, outcome measures and study designs were often highly heterogeneous, even within a single initiative. As a result, relevant data are presented in tables with accompanying narrative summaries as appropriate. Also, given the diversity and heterogeneity of initiatives, no attempt was made to compare studies across intervention categories. However, where studies related to more than one category, links and cross-referrals were made between reviewers and across the reports of their reviews.

Formal assessment of study limitations was not undertaken.^11^ Evidence was categorised according to the underpinning study design (tier 1: review; tier 2: primary research studies). Within this, it should be recognised that many studies were not specifically designed to evaluate the outcomes of interest, which were often only documented within studies as a secondary outcome.

Public involvement was elicited at several stages of the project. In January 2024, members of the standing public advisory group for the Sheffield ESG advised on the Plain Language Summary for the project website. Once analysis of the evidence base had been completed in late March 2024, members of the School’s Patient and Public Involvement panel were invited to submit their responses to questions on the relevance of the measures and the rationale of the interventions. The review team drew on these responses when interpreting the findings.

## Results

**Figure 1:**
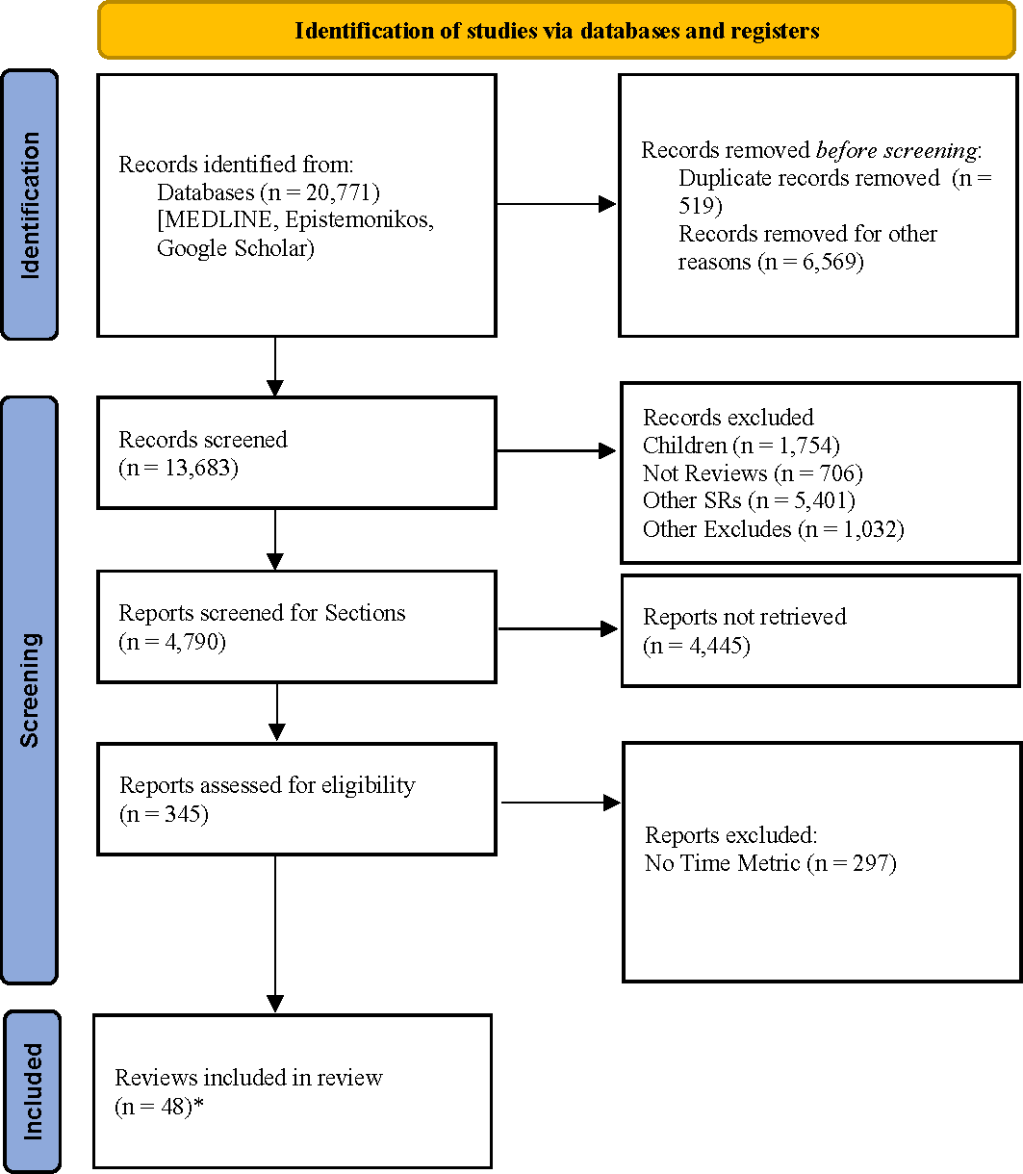
PRISMA diagram: Tier 1 review-level evidence. PRISMA, Preferred Reporting Items for Systematic Reviews and Meta-Analyses; SRs, systematic review. *10 reviews appear under more than one initiative. *From:* Page MJ, Mc Kenzie JE, Bossuyt PM, Boutron I, Hoffman TC, Mulrow CD, et al. The PRISMA 2020 statement: an updated guideline for reporting systematic reviews. BMJ 2021;372:n71. doi:10.1136/bmj.n71

The search for review-level evidence, plus the search for studies referencing the outcomes of overcrowding, ambulance response and waiting times, yielded a total of 20 771 citations. The review team then searched within the EndNote database for specific terms generated by the team or supplied by NHS England and assigned any retrieved results to the relevant folder for each review. When additional terms were identified, records matching these terms were added to that folder for screening. This approach optimised the search process within the available time frame while keeping the search strategy responsive to subsequent refinement, recognising the diffuse terminology being used. After checking full texts, a total of 48 reviews met the criteria and were used across the 10 sections with 10 reviews being cited in more than one section. The results of this literature search and screening process are summarised in the PRISMA flow diagram (figure 1).

For 7 of the 10 initiatives, the quantity of tier 1-level evidence was deemed insufficient, so supplementary searches were conducted for tier 2 evidence. The results of the full search and study selection processes are presented in table 3.

**Table 3:** Results of search and study selection process across all 10 high-impact initiatives

High-impact initiative	Tier 1—review articles					Tier 2—experimental/observational studies				
	Title/Abs (n)	Full text (n)	Includes (n)	Includes from other sources (n)	Final includes (n)	Title/Abs (n)	Full text (n)	Includes (n)	Includes from other sources (n)	Final includes (n)
Urgent community response	100	32	3	2	5					
Same day emergency care	10	2	0	1	1	1093	39	5	1	6
Acute frailty	46	19	2	1	3	131	57	3	0	3
In-patient flow	475	135	22	11	33					
Care transfer hubs	100	29	1	1	2					
Community beds	41	15	0	0	0	81	8	0	0	0
Intermediate care	49	21	3	0	3	1318	196	0	0	0
Single point of access	6	0	0	7	7	391	22	6	0	6
Acute respiratory infection hubs	256	32	1	0	1	279	11	0	0	0
Virtual wards	153	60	2	1	3	136	84	1	0	1
Total					58*					16

* 10 reviews appear under more than one initiative.

Some of the high-impact initiatives hold very precise meanings (eg, SDEC, acute respiratory infection (ARI) hubs). In contrast, other initiatives cover very heterogeneous interventions that share little in common (eg, under the headings of urgent community response (UCR) and in-patient flow). Also, the categories for the high-impact initiatives were not mutually exclusive, with overlapping interventions, reviews and cited studies. In general, the 10 initiatives had not been evaluated against the ambulance response metric, although proximal measures such as ambulance turnaround times and ambulance offload delay did receive coverage in the research literature. However, ambulance-related outcomes were reported only for 4 of the 10 initiatives. ED wait times (and the related outcome, ED LOS, which has been linked to mortality),^2^ were more commonly studied (data were available for 9 of the 10 initiatives). One might still expect to see an indirect impact on these outcomes by the initiatives, despite, as noted above, the principal rationale for most initiatives being to affect hospital bed occupancy rates. However, it proved challenging to identify studies where the two focal metrics were being evaluated by relevant interventions. Summaries of the quantity of evidence and findings for each initiative together with their implications are presented in onlinesupplemental appendix 4^5^.

### Urgent community response

Five reviews^12^,^16^ reported relevant findings from only five primary research studies from the UK, Sweden and Canada with very limited evidence that such interventions might reduce either ambulance mission and duty times or offload delay.^12 15^ Four reviews reported on ED wait times/LOS, with three reporting a beneficial effect^13 15 16^ and one a negative effect.^14^

### Same day emergency care

One review^17^ reported relevant findings from two primary research studies, supplemented by four additional primary research studies. All six studies examined UK-based SDEC initiatives, to which a varying proportion of select patients were referred from the ED, ranging from as few as 8% of SDEC patients^18^ to all SDEC patients,^19 20^ where reported. All studies reported reduced ED wait times, either as reductions in the number exceeding the 4-hour target,^18^ higher proportions achieving this target, or much lower wait times generally, in the SDEC than in the ED^19 21^ or across the department as a whole,^20^ or as non-comparative studies reporting SDEC wait times that fell within the 4-hour target.^22 23^ There was no evidence of ambulance response times or similar metrics.

### Acute frailty

Three reviews,^24^,^26^ each reporting findings from between one and six relevant primary studies from an international evidence base, including the UK, as well as three additional primary research studies—a Finnish RCT^27^ and observational study^28^ and a UK pilot service evaluation^29^—all of which reported on ED wait times or ED LOS. Overall, review-level and primary research evidence indicated a reduction in ED wait time or a trend towards shorter wait times, although study-level findings were sometimes reported incompletely at the review level or as an abstract only publication. The evidence base related to older adults and to interventions within the ED only, including acute frailty assessment zones and different staffing models of care. Some interventions specifically emphasised the facilitation of early assessment. Staffing models varied by clinical lead, types of assessment and multidisciplinary team support. There was no evidence found on ambulance response times.

### In-patient flow interventions

33 reviews covering a large number of relevant primary studies (n≥200) from an international evidence base. The included interventions were broadly categorised as teams or roles-based, pathways or specific patient groups, units, technology, bed management, point-of-care testing, target-based, protocols and standardisation of processes, and other. The reported impact of the interventions under each category on ED wait times was generally inconclusive: a majority of included studies in any review might report finding in favour of interventions, for example,^30^,^32^ but some reviews reported studies that found no difference between interventions and previous practice,^33^ and even reported some increase in ED wait times with the intervention.^31 34^ There was no evidence on ambulance response times. Ambulance offload delay was examined in one review of at least 10 different interventions,^35^ which generally noted a reduction in offload delay times.

### Care transfer hubs

Two reviews, reporting relevant findings from five primary research studies from the UK, the USA, Belgium and Australia, evaluated the impact of diverse ‘transitional care interventions’ (TCIs) implemented within the ED.^36 37^ One review reported a clear reduction in ED wait times for a multidisciplinary team-led intervention,^36^ but the other review reported inconclusive findings for a nurse-led intervention.^37^ There was no evidence on ambulance response times.

### Community beds

No relevant studies were identified.

### Intermediate care

Intermediate care provides one example of where intervention categories overlapped with each other; in this case TCIs (as also included within care transfer hubs). Three reviews^36^,^38^ each with three or more relevant primary research studies from an international evidence base (including the UK), reported the impact on ED wait times of either TCIs^36 37^ or telestroke services^38^ in older patients. Two reviews reported evidence of a reduction in ED wait times^36 38^ while the findings of the third review were inconclusive.^37^ There was no evidence on ambulance response times.

### Single point of access

Seven reviews with primary research studies from Europe (including the UK), Australia and New Zealand found that integrating primary care services or providers within or alongside EDs^14 39^ or the availability of short-stay mental health crisis units^30 40^ might potentially reduce ED LOS for certain patient groups. However, the impact was not consistent across all settings.^41 42^ One review (with 21 relevant primary research studies from an international evidence base, including the UK) found that using general practitioners (GPs) within an emergency medicine setting reduced ambulance duty cycles and conveyance rates.^12^

### ARI hubs

One review^43^ with four relevant primary research studies from the USA and Canada reported that a specific ARI intervention in a UEC setting reduced ED wait time. There was no evidence on ambulance response times.

### Virtual wards/hospital at home

Three reviews (which included 18 relevant primary research studies) evaluated prehospital telemedicine interventions: telepsychiatry,^38^ telestroke^44^ and ambulance-based telemedicine for general prehospital emergency populations^45^ and found that these interventions reduced ambulance response times or ‘time to treatment’. One review,^38^ with one relevant primary research study from the USA evaluating telepsychiatry and one primary study from the USA evaluating hospital at home (HAH),^46^ each reported that these interventions reduced ED wait time or ED LOS.

## Discussion

This review offers a broad overview of the 10 high-impact interventions identified by NHS England and their potential impact on two metrics. The evidence suggests that some initiatives, such as SDEC, acute frailty services, care transfer hubs with multidisciplinary teams and telestroke services may effectively reduce ED wait times or ED LOS. However, the impact of in-patient flow interventions on this outcome was generally inconclusive, with some studies reporting reductions and others showing no difference or even an increase. Regarding ambulance response times, there was limited evidence, with only a few interventions, such as integrating GPs within emergency medicine settings and prehospital telemedicine interventions, showing potential benefits. For ambulance offload delays, one review found that various interventions generally reduced offload delay times, but the evidence was limited. This review highlights the need for research to establish the effectiveness of these interventions in different settings and contexts.

The principal limitations affecting the evidence base concern both the categorisation of interventions and the headline outcomes used by NHS England. In terms of the interventions, initiatives such as SDEC, ARI hubs and the HAH component of ‘virtual wards’, had sufficiently robust definitions and ‘name recognition’ to facilitate targeted evaluation within the research literature, although there was still variation in the services delivered under these three initiatives. The remaining initiatives all exhibited a much higher degree of uncertainty in how key elements of the interventions might be defined. This, in turn, made it challenging to identify relevant interventions within the published literature, even allowing for the use of an extensive list of potential intervention synonyms (see table 2). Some initiatives included multiple, often indistinct interventions, that appear to hold little in common beyond their overall intent (eg, UCR or in-patient flow). A review based exclusively on the 10 ‘high-impact initiatives’ as itemised by NHS England would have returned very few studies. For example, NICE guidance on the effectiveness of ARI hubs failed to identify any specific published evidence at all.^47^ However, even an initiative like SDEC might encompass a variety of service differences, and this heterogeneity of interventions and intervention types, both within and across initiatives, also increases the uncertainty regarding some findings and limits their generalisability to different contexts.

The two NHS England ‘headline metrics’ were ED wait times and ambulance response times. In terms of the research identified for this review, ED wait times and potentially related metrics (such as ED LOS) were reported as primary or secondary outcomes for nine of the 10 initiatives (only community beds had no evidence). ED LOS, while different from ‘wait time’ or ‘time to be seen’, has been included here to capture a more extensive evidence base and because it is a known potential predictor of important outcomes, such as mortality.^2^ Ambulance response times and other time-related metrics for ambulances were not evaluated at all for 6 of the 10 initiatives. The remaining four initiatives measured outcomes such as mission or duty time for UCR, offload delay for some in-patient flow interventions, duty cycles and conveyance rates for single point of access and metrics such as ‘onset to door’ time (within ‘virtual wards’ for prehospital telemedicine). Given how the majority of these interventions work or are envisaged to work, the absence of evidence on these two outcomes is not surprising. ‘Ambulance response times’ might only apply to a small number of high-impact initiatives while ‘ED wait times’ fail to recognise the whole-system impact of alternative high-impact interventions. As a consequence of this review’s focus on specific outcome metrics, few of the thousands of reviews and primary research studies screened for the period of this review (2018–2024) were adjudged relevant to our particular review question. The evaluated interventions commonly focused on outcomes such as ED visits, admissions, ‘overcrowding’, ‘ambulance offload delay’ and ‘inpatient flow’. While ambulance response times and ED wait times constitute important ‘headline’ metrics for emergency care, they are not typically either the first or most informative choice when evaluating the impact of many urgent care initiatives. Therefore, it should be noted that this review did not aim to evaluate all of the potential effects or wider benefits of the initiatives. A review of other metrics, such as admission/utilisation and discharge rates, might produce quite different findings. This highlights the limitations of seeking to apply a limited number of headline metrics across all emergency care contexts.

While the diversity of ‘high-impact initiatives’ from the specific to the broad, from the clearly defined to the diffuse, and from the hospital based to the community focused, makes an even and consistent approach to evidence gathering and assessment challenging, this review made great efforts to identify available evidence to a level appropriate for each initiative and any related interventions. As such it offers a valuable compendium. Notwithstanding such limitations, clear patterns to findings can still be observed. For example, in terms of reduction in ED wait times, there is consistent primary study level evidence for a clearly defined initiative such as SDEC, or generally consistent review-level evidence for diverse in-patient flow initiatives, despite both approaches exhibiting high variability in terms of what service is offered, together with context-sensitivity by particular centre (online supplemental file 4). However, it is also worth noting that a SDEC unit might also simply involve the provision of more staff and resource elsewhere within the ED setting, such as an additional senior decision-maker,^20^ who might have had a similar impact if provided within the ED itself. The evidence base supporting the 10 high-impact initiatives might, therefore, be very limited for the outcomes of interest, but this review does offer a broad overview of the potential evidence for 10 high-impact interventions identified by NHS England.

If future research is to effectively inform policy in this field, then it needs to be high quality (in design, conduct and reporting), be conducted over adequate periods of time and target clearly defined interventions that satisfy the identifiable characteristics (and activating mechanisms) of known real-world interventions, such as the 10 initiatives. It needs to measure outcomes specified as important by policy-makers, professionals, patients and the public, as well as those that are directly relevant to each initiative and how it is intended to work (ie, which outcomes it targets directly). Alternatively, research is needed that demonstrates a clear link between outcomes that are frequently measured for interventions (such as ED visits) and those outcomes of particular interest to policy-makers, for example, shorter ED wait times. This confirms what the review team were told by both clinical experts and patient representatives: wider, more contextually sensitive communication around initiatives and their evaluation is required.

This scoping review has limitations. The work was originally designed as a systematic review of reviews, but a scoping review was performed. This was because the evidence proved so limited in some instances, and so extensive in others, that broader processes had to be applied, including searching for additional evidence (both primary research studies and the extensive complementary searches for studies based on outcomes), and the use of single reviewers for the review of some initiatives. Second, within this context, a decision was also taken to limit the number of bibliographic databases searched, as long as it included MEDLINE/PubMed because it is known to have excellent coverage of UEC.^48^ Finally, as a scoping review of many different study designs, quality assessment of the evidence base was not performed; this also needs to be considered when reflecting on the findings.

## Conclusions

Identifying research evaluations of the named high-impact initiatives, or interventions that share their characteristics, was extremely challenging. Few studies were found to cover the impact of relevant or potentially relevant interventions on the target outcomes of ED wait times and ambulance response times. While some evidence suggests that some ‘high-impact initiatives’, such as SDEC, acute frailty units, care transfer hubs and some in-patient flow interventions might reduce ED wait times or LOS; and weak evidence suggests a beneficial effect for UCR, prehospital telemedicine and some in-patient flow interventions on some time-based outcomes for ambulances, the evidence base overall is limited in both quantity and quality. As a result, continuing uncertainty surrounds the efficacy of these specific initiatives in relation to these outcomes of interest. High-quality research focused on clearly defined interventions that satisfy the identifiable characteristics of the 10 initiatives remains a priority.

## supplementary material

10.1136/bmjoq-2024-002906 online supplemental file 1

10.1136/bmjoq-2024-002906 online supplemental file 2

10.1136/bmjoq-2024-002906 online supplemental file 3

10.1136/bmjoq-2024-002906 online supplemental file 4

10.1136/bmjoq-2024-002906 online supplemental file 5

## Data Availability

All data relevant to the study are included in the article or uploaded as online supplemental information.
