# Supplementary material for: Scoping review of the effectiveness of 10 high-impact initiatives (HIIs) for recovering urgent and emergency care services
Source: BMJ Open Qual. 2024 Sep 18;13(3):e002906. doi: 10.1136/bmjoq-2024-002906 (PMC11429364; doi:10.1136/bmjoq-2024-002906)
Supplement: online supplemental file 1 [file bmjoq-13-3-s001.pdf]

Appendix 1: The 10 High Impact Initiatives

This information was compiled by the authors for the report commissioned by NHS England.

Initiative	Description	Related/included initiatives and goals
Urgent community response	Urgent Community Response refers to initiatives that are aimed at providing fast and efficient urgent care at home and on-site within the community. Urgent Community Response is limited to community-based urgent care services (unlike those co-located with the ED; see Single Point of Access). Also note that community-based high impact interventions that aim to reduce ED readmissions among recently discharged patients are reviewed separately (see Care Transfer Hubs and Community Beds), as are those involving home-based hospital services (see Virtual Wards).<br><br>Some key features of Urgent Community Response initiatives include:<br>— Aimed at long-term care patients as well as the general population;<br>— Varied in nature; care may be provided by GPs, community paramedics, and other healthcare professionals;<br>— Often available outside the usual hours of care, such as on evenings and weekends;<br>— Urgent care may be provided in the patient's home or in clinics or walk-in centres;<br>— Ongoing referral to ED provided in cases where hospital treatment is required;<br>— Tailored to the needs of local communities;<br>— More cost-efficient and better patient satisfaction when ED admittance can be avoided	Urgent Community Response includes:<br>— Rapid Response Service (RRS): providing urgent care to patients at home rather than in hospital, operating 24 hours a day and 7 days a week to support adult patients with acute illnesses;<br>— Hospice rapid response service (HRRS): providing urgent care to residential patients, may work collaboratively with domiciliary staff in hospices and may include provision of palliative care;<br>— Urgent home care and Community-based urgent care service: targeting older people and adults with complex health needs who require urgent medical attention that can be provided at home rather than in hospital; may involve integrated care with a range of services and health care professionals;<br>— Out-of-hours care, after hours care and walk-in services operating outside of normal working hours;<br>— Services equipped to treat minor injuries and refer to ED if required; non-traditional pathways include protocol-driven referrals to radiography clinics and district nurses<br><br>The goal is to improve patient care in the community, where possible enabling patients to stay in their own home, avoiding the need for ambulance services and hospital admission.
Same Day Emergency Care (SDEC)	Same Day Emergency Care (SDEC) or Ambulatory Emergency Care (AEC) is a type of healthcare facility that provides walk-in, extended-hour access to medical care for injuries or illnesses that require immediate attention but are not life-threatening emergencies. It has been developed within the NHS (NHS England 2018), as mandated in 2019 by the NHS Long Term Plan (NHS England, 2019); and has a specific strategy: The Same Day Strategy for SDEC (NHS England, 2021). The aim is to reduce the pressure on the emergency department (ED) by providing a specific emergency service for patients who are likely to be discharged within 12 hours and who, if necessary, can attend again on subsequent days, for	Related/included initiatives: SDEC is a specific approach to Ambulatory Emergency Care (AEC), with a specific strategy.<br><br>The goal is to provide convenient, affordable access to quality medical care for acute needs that are unlikely to result in a hospital admission, thus reducing patient numbers within the ED and hospital admissions.

	further investigation or review, rather than being admitted (Atkin 2022, Dean & Barratt 2024).<br><br>Some key features of SDEC/AEC include:<br><br>— Patients can be referred to SDEC treatment through different routes, including: Following streaming or triage in emergency departments (EDs); Direct referral from GPs; Direct transfer from ambulance services; Direct referral from NHS 111;<br>— SDEC treatments include: Medical; Surgical; Specialist (e.g. Paediatrics and Gynaecology etc.);<br>— Staffed by a multidisciplinary team, but with an identified senior clinical decision-maker on duty during opening hours;<br>— Low or very low likelihood of patient admission, or patients satisfy criteria to be discharged and managed at home or with outpatient appointments, rather than admission	
Acute frailty	Acute frailty services 'refer to those services that identify and respond to the needs of frail, usually older people presenting to urgent and emergency care (UEC) services' (NHS England 2019). While no single age-related definition exists for frailty, it is a condition associated with 'a cumulative decline of physiological resilience across several body systems' (Jarman 2021). Commissioning for Quality and Innovation (CQUIN) included the identification and response to frailty in the ED, specifying comprehensive geriatric assessment (CGA) and/or referral to the acute frailty service for those patients with a Clinical Frailty Scale (CFS) score of at least six (CQUIN 2023). As part of the NHS Long Term Plan, the FRAIL strategy further highlights a commitment to every NHS hospital in England having an 'acute frailty service/same day emergency care service' in operation for 70 hours per week, supporting safe discharge and avoiding overnight admissions (NHS England 2024). Models of care may integrate validated frailty assessment tools with other considerations for optimal service delivery, such as the physical environment and clinical staffing of urgent and emergency care services.<br><br>Some key features of Acute Frailty services include:<br><br>— Triage tools for frail adults in the ED (including triage for frailty and among frail adults);<br>— Acute frailty assessment area/zone in the ED	Related/included initiatives and tools: Acute frailty assessment area; acute frailty zone; Clinical Frailty Scale (CFS) and Clinical Frailty Scale 9 (CFS9); Emergency Severity Index in the ED; FRAIL scale; Geriatric ED Improvement taskforce; Geriatric Emergency Department Intervention model (GEDI); Geriatric Emergency Department Innovations/Geriatric Nurse Liaison (GEDI/GNL) in the ED; Geriatric Emergency Medicine Service model (GEMS); Geriatrician-led Comprehensive Geriatrics Assessment (CGA); geriatric liaison team (GLT); InterRAI ED Screener; Mater Aged Care in An Emergency service (MACIAE); National Early Warning Score 2 (NEWS2); OPAL team (older people assessment and liaison); Program of Research to Integrate Services for the Maintenance of Autonomy (PRISMA7); Silver Code (SC) prognostic tool; systematic geriatric assessment; three-level ED triage scale; Trauma Specific Frailty Index (TSFI); (URGENT).<br><br>The goal is for acute frailty services to improve the triage of older adults at risk of, or with frailty, and thereby improve emergency department (ED) wait times and related outcomes.

	— Staffing models of care for frailty in the ED	
In-patient flow	Numerous and diverse interventions can improve inpatient flow within Urgent and Emergency Care Systems (UECS). Triage and assessment are typically key features of the management of inpatient flow; for the purposes of this review 'triage only' interventions are excluded although triage, and associated activities such as streaming patients to the most appropriate service (e.g., ED, urgent care, primary care) based on their needs, form a component of many separate interventions.<br><br>Collaboration between UECS and Community Services may involve other high impact interventions, such as Urgent Community Response and Care Transfer Hubs. Some key features of in-patient flow interventions include:<br><br>— Interventions to reduce ED exit block (e.g. full capacity protocols, escalation protocols - alongside discharge planning and coordination which is the main way of improving in-patient flow.<br>— To include greater staff and patient involvement, and use of estimated discharge dates).	Related/included initiatives: Other approaches that target inpatient flow include diagnostic services such as point-of-care testing (POCT), short stay units, early discharge programmes and bed management systems. Interventions that operate at an organizational and administrative level include increased staffing and referral pathways. Another related intervention is the use of kiosks and patient self-scheduling which allow patients to book appointments for non-urgent conditions. Interventions for "boarding" were specifically excluded from this review.<br><br>The goal of tackling inpatient flow in emergency departments (EDs) is to achieve a smooth and efficient patient experience. This is achieved through reduced crowding and wait times, increased bed availability and optimized staff utilization: By streamlining processes, interventions can help staff focus on patient care rather than managing logistical bottlenecks.
Care Transfer Hubs	Care transfer hubs constitute social and healthcare multi-level coordinating centres (virtual or in-person) that are (partially or fully) located at acute hospital sites.<br><br>The main aims of care transfer hubs are: i) to assist with patients' discharge, ii) to promote patients' recovery, and iii) to reduce acute hospital admission rates. To do so, care transfer hubs link all the relevant services across settings (i.e., acute, community, primary care, social care, housing and voluntary) to provide coordinated care (NHS, 2024). Care transfer hubs also support patients who require short-term community bed-based rehabilitation recovery or assessment of long-term needs.	Related/included initiatives: Discharge coordination teams; Transitional care teams; Virtual discharge teams; Community discharge teams; transfer of care hub; discharge hub/team/cell/; integrated discharge hub/ team/ service; single point of access; home first hub/centre; coordination hub/centre.; home safe; multi-agency hub; transfer care bureau; care point; community assessment team; discharge command centre; front door and hospital discharge; health and social care hub; intermediate care assessment team; onward care team; right care<br><br>The goal of care transfer hubs are: i) to develop timely and person-centred "step-down" or "step-up" plans for patients, ii) to support early discharges, iii) to define which pathway each patient should be placed on, iii) to ensure patients' safety and welfare through multidisciplinary coordinated care (NHS, 2024).
Community beds	In the urgent and emergency setting, a community bed refers to a bed-based facility typically found in various settings, including community-	Related/included initiatives: Step-down beds; Transitional care beds; Intermediate care beds; Rehabilitation beds; Community recovery beds

	commissioned hospitals, acute hospitals, community hospitals, residential care homes, care homes with nursing services, standalone community bed-based intermediate care facilities, independent sector healthcare provider facilities, local authority facilities, or other bed-based settings. These beds provide short-term care, offering assessment and clinical interventions to patients within their local area.<br><br>Some key features of a community bed include:<br>— Provides care in a bed-based setting;<br>— Mainly used for short-term observation, assessment, or treatment;<br>— Includes a multidisciplinary health and/or social care and support	to include local authority and NHS maintained beds; P2 beds; Discharge to Assess (D2A) beds.<br><br>The goal is to prevent unnecessary hospital admissions for patients who need ongoing care but not full hospitalisation, to reduce long-term care admissions and to support timely discharge by implementing in-hospital efficiencies and bringing forward discharge processes.
Intermediate care	Intermediate care constitutes a type of healthcare service that aims to promote patients' independence by assisting them with recovery after discharge from acute inpatient services or virtual wards (NICE, 2024). The core principles underpinning intermediate care services are (NICE, 2017): i) to adopt a person-centred approach that promotes patients' independence and enhance their well-being, ii) to establish an optimal communication channels among intermediate care practitioners, agencies, patients and their families/carers, iii) to focus on patients' individuals strengths, iv) to ensure that patients and their families/carers can gain direct access to intermediate care services and are provided with all the information to do so.<br><br>Intermediate care can be multicomponent (i.e., health care-based, social care-based, or combined) and can be delivered by multi-disciplinary teams (e.g., therapists, nursing staff, social workers) either at patients' home (home-based, discharge pathway one) or community bed settings (e.g., hospital; bed-based, discharge pathway two) (i.e., step-down intermediate care) (NHS, 2023). Four stages underpin the implementation of intermediate care services (NICE, 2024): i) the initial stage where a professional conducts the first assessment and establish a contact with the patients or their families/carers, ii) the second stage where the service is implemented and can last from a few hours to a few days, iii) the third stage where patients' progress is re-evaluated, and iv) the fourth stage when the intermediate care service is complete and the patient is transferred to another service or be provided with all the information in case they need to attend the service in the future.<br><br>Diverse intermediate care services are provided with the most common being: reablement, crisis response, home-based, bed-based (NICE, 2024).	Related/included initiatives: relevant intermediate care terms, such as community hospital, residential care home, nursing home, stand-alone intermediate care facility, independent sector/local authority facility, step-down, (non)bed-based settings, patients' home-based, person-centred approach/risk-taking, and reablement, were all considered eligible for inclusion in this review.<br><br>The main goals of intermediate care services are (NICE, 2024): i) to assist patients, who are in increased difficulty due to illness or disability, in remaining at home, ii) to assist patients with their recovery (e.g., after a fall, acute illness, or operation), iii) to reduce the likelihood of unnecessary hospital visits, and iv) to expedite patients' discharge after a hospital stay. Intermediate care services can include any adult irrespective of their age, with a particular focus on those who are at-risk of admission to residential care (individuals with dementia or mental health needs are also included) and can integrate with other health and social care services (NHS, 2023).

	Given that intermediate care services permeate the whole discharge process, any intervention, programme, or strategy implemented at pre-discharge, during the discharge process, and post-discharge were considered eligible for inclusion in this review (including transition programmes).	
Single Point of Access (SPoA)	In the context of this review, a "single point of access" (SPoA) refers to a centralized service that acts as a single entry point for patients seeking urgent or emergency care services. The SPoA model is being adopted by various regions and healthcare systems across England, as a response to the UEC Recovery Plan, to provide a streamlined and efficient access point for patients seeking urgent or emergency care.<br><br>Overall the term "Single Point of Access" is unsatisfactory as it can include diverse models whether in the hospital or in the community. One of the most commonly-investigated SPoA models involves location of a primary care health professional as the front door or first port of call. Mental health crisis centres also serve as a single port of call. Arguably NHS 111 is also based on a single point of access. Finally, NHS England has promoted a specific interpretation of "Single Point of Access" that is not captured at all within the research literature, but which has been successfully rolled-out as a common model of alternative care provision across the NHS. In this context there is some overlap with review 1 on Urgent Community Response. However, a Single Point of Access is expected to provide access to diverse services, including emergency care and not just alternative community provision. In contrast Urgent Community Response is expected to be largely self-sufficent except in the case of actual emergencies.<br><br>Generically, the NHS 111 service is predicated on a single point of access model and much use of the "single point of access in the last decade or so has foregrounded that interpretation. Further distinctions may be observed in the location of a face-to-face SPoA in the community or alongside/ within a hospital campus. Provision of telephone or other remote services offer the prospect of a location-independent SPoA. SPoAs may be staffed by health professionals or by administrative staff in the role of navigators.	As the Single Point of Access model is defined by the presence of a single component, numerous other synonyms or variants are covered broadly by the model; (e.g. Integrated Urgent Care (IUC) Service; Urgent Care Treatment Center (UCTC); Urgent Treatment Center (UTC); Coordination Hub; Clinical Navigation Hub; Clinical Advisory Service; Clinical Assessment Service (CAS); Multidisciplinary Clinical Assessment Service; Acute Integrated Care Hub; Integrated Care Hub; Urgent Care Center (UCC); Urgent Emergency Care Service; Urgent Care Advice Line; Single Call Access Point; Unified Access Point (UAP); Integrated Urgent Care Access (IUCA); Front-door Access (FDA); Centralized Access to Care (CAC); Single Entry Point (SEP); Single Point of Triage).<br><br>The main objectives of a single point of access are:<br><br>— To streamline access to care so that patients access various services through a single contact point, such as a phone number or online portal, rather than navigating multiple entry points.<br>— To provide triage and coordination whereby trained professionals (e.g., nurses, paramedics) assess the patient's condition, provide initial advice, and direct them to the most appropriate care setting or service based on their needs.<br>— To reduce unnecessary hospital admissions by triaging patients and directing them to the right level of care, the SPoA aims to reduce.<br>— To improve patient flow different urgent and emergency care services whether emergency departments, urgent care centres, ambulance services, or community-based services.

	However, it is generally agreed that time to contact with a health professional is often critical to the effectiveness of the service.<br><br>Fast track services may utilise an SPoA model but they are explicitly excluded from this review as not specific to the “high impact response”.	
Acute Respiratory Infection Hubs (ARI hubs)	An acute respiratory infection (ARI) hub is typically a dedicated facility or area within a healthcare system that is specifically designed to manage and treat patients with suspected or confirmed acute respiratory infections during periods of high prevalence, such as during flu seasons or respiratory disease outbreaks. The specific activities and operations of an ARI hub may vary depending on the healthcare system, the nature of the respiratory infection outbreak, and the available resources and infrastructure. Acute respiratory infection (ARI) hubs constitute a healthcare service embedded within the national health system that aims to provide timely care to children and adults with respiratory conditions and reduce the pressure on the other parts of the healthcare system (NHS, 2024).<br><br>Typical functions of an ARI hub may include:<br><br>— Triage and screening: The ARI hub would have a designated triage area to screen and assess patients with respiratory symptoms, separating them from other patient populations to minimize the risk of transmission.<br>— Testing and diagnosis: The hub would be equipped with the necessary resources for testing and diagnosing respiratory infections, such as rapid diagnostic tests, chest X-rays, and laboratory facilities for processing samples.<br>— Isolation and treatment: Patients with confirmed or suspected acute respiratory infections would be isolated and treated in dedicated areas within the ARI hub, following appropriate infection control protocols and using personal protective equipment (PPE) as necessary.<br>— Respiratory support: Depending on the severity of the infections, the ARI hub may have specialized equipment and personnel for providing respiratory support.<br>— Monitoring and management: Healthcare professionals within the ARI hub would closely monitor the condition of patients, manage their symptoms, and provide appropriate medical interventions.	Related/included initiatives: ARI hubs, respiratory infection hubs, respiratory care clinics, respiratory assessment centres, upper respiratory infection clinics, ARI centres, paediatric ARI hubs<br><br>The main goals of ARI hubs are (NHS England, 2024):<br><br>— To facilitate same-day access to assessment and specialist advice for those with urgent clinical needs.<br>— To reduce ambulance callouts, A&E attendances, and hospital admissions.<br>— To free up workforce resources (e.g., time for GP practice teams).<br>— To provide accessible and equitable service to support same day access.<br>— To reduce infection spread in GP practices’ waiting rooms and clinics and emergency departments (ED).

	— Coordination and communication: The ARI hub would coordinate the response to respiratory infection outbreaks, communicating with public health authorities, and managing allocation of resources and personnel.<br>— Surge capacity planning: During periods of high demand, the ARI hub may implement surge capacity plans, such as expanding bed capacity, staffing, or establishing temporary treatment facilities.<br><br>Other activities of an ARI hub may include surveillance – through collaboration with public health agencies to study the epidemiology, transmission patterns, and clinical characteristics of emerging or prevalent respiratory infections – and patient education in providing resources to patients and their families on self care and infection control measures.	
Virtual wards	A virtual ward is a type of healthcare facility that provides acute medical care in the patient's home instead of in a hospital, for injuries or illnesses that require attention but are not life-threatening emergencies. It is also commonly known as hospital at home (HAH). NHS England launched its virtual wards programme in April 2022 and is continuing to publish guidance on specific aspects of virtual wards or specific populations (NHS England, 2024).<br><br>Some key features of virtual wards (or HAH) include:<br>— Short-stay (up to 14 days), acute-only services for people who would otherwise be in hospital;<br>— They can provide either 'step-up' or 'step-down' care treatments;<br>— Patients should have access to the same services they would in hospital, including urgent diagnostics, blood tests, intravenous therapy, 24-hour management from a multidisciplinary team and face-to-face meetings with specialists;<br>— Virtual wards should be technology enabled, with clinicians, patients and carers making decisions together about the use of available technologies based on clinical appropriateness and patient/carer preferences	Related/included initiatives: Virtual wards or domicilary care; Hospital at Home (HAH); Remote patient monitoring (RPM); Telehealth care (includes both RPM and prehospital telemedicine in an emergency medical context (e.g. using technology for consultation purposes when attending patients in the community))<br><br>The goal is to provide convenient, affordable, personalised care for acute needs that can be managed outside of hospital, thus reducing pressure on hospital beds, increase workforce efficiency and inpatient flow, and reduce likelihood of some of the risks associated with an in-hospital stay (Chappell et al, 2024).

ED: Emergency Department; iUEC: Integrated Urgent Emergency Care; NHS: National Health Service

References:

ATKIN C, KNIGHT T, COOKSLEY T, HOLLAND M, SUBBE C, KENNEDY A, VARIA R, GEBRIL A, LASSERSON D. Performance of admission pathways within acute medicine services: Analysis from the Society for Acute Medicine Benchmarking Audit 2022 and comparison with performance 2019 - 2021. Eur J Intern Med. 2023 Dec;118:89-97

CHAPPELL P, CO M, HARDIE T, LLOYD T, TALLACK C, GERHOLD M, MAYERS C. What do virtual wards look like in England? (Working Paper). Health Foundation; 2024 (https://www.health.org.uk/publications/what-do-virtual-wards-look-like-in-england).

CQUIN; 2023. Commissioning for Quality and Innovation (CQUIN): 2023/2024 Guidance. Available at: 2023-24 CQUIN guidance (england.nhs.uk) [accessed 25.03.24]

DEAN, S. & BARRATT, J. What is the existing evidence base for adult medical Same Day Emergency Care in UK NHS hospitals? A scoping review, Future Healthcare Journal (2024), doi: https://doi.org/10.1016/j.fhj.2024.100011

NHS ENGLAND (2021). Same Day Strategy. https://www.england.nhs.uk/long-read/sameday-strategy/

NHS ENGLAND (2022). Urgent Community Response https://www.england.nhs.uk/community-health-services/urgent-community-response-services/

NHS ENGLAND (2023). Intermediate care framework for rehabilitation, reablement and recovery following hospital discharge [Online]. Available: https://www.england.nhs.uk/publication/intermediate-care-framework-for-rehabilitation-reablement-and-recovery-following-hospital-discharge/ (Assessed 09 Mar. 2024).

NHS ENGLAND (2024). Combined adult and paediatric acute respiratory infection (ARI) hubs [Online]. Available: https://www.england.nhs.uk/long-read/combined-adult-and-paediatric-acute-respiratory-infection-ari-hubs/ (Assessed 14 Mar. 2024).

NHS ENGLAND 2018. NHS Improvement and the Ambulatory Emergency Care Network. Ambulatory emergency care guide: Same day emergency care: clinical definition, patient selection and metrics. 2018. https://www.england.nhs.uk/urgent-emergency-care/same-day-emergency-care/ambulatoryemergency-care-guide-same-day-emergency-care-clinical-definition-patient-selection-and-metrics/

NHS ENGLAND AND NHS IMPROVEMENT (2019). The NHS Long Term Plan. https://www.longtermplan.nhs.uk/publication/nhs-long-term-plan/

NHS ENGLAND, 2024: Virtual Wards https://www.england.nhs.uk/virtual-wards/

NHS ENGLAND; 2019. Same-day acute frailty services. Available at: SDEC_guide_frailty_May_2019_update.pdf (england.nhs.uk) [accessed 25.03.24]

NHS England; 2023. Delivery plan for recovering urgent and emergency care services. Available at: NHS England » Delivery plan for recovering urgent and emergency care services [accessed 25.03.24]

NHS England; 2024. FRAIL strategy. Available at: NHS England » FRAIL strategy [accessed 25.03.24]

NICE (2017). Intermediate care including reablement [Online]. Available: https://www.nice.org.uk/guidance/ng74 (Assessed 09 Mar. 2024).

NICE (2024). Understanding intermediate care, including reablement [Online]. Available: https://www.nice.org.uk/about/nice-communities/social-care/quick-guides/understanding-intermediate-care (Assessed 09 Mar. 2024).
