# Supplementary material for: Scoping review of the effectiveness of 10 high-impact initiatives (HIIs) for recovering urgent and emergency care services
Source: BMJ Open Qual. 2024 Sep 18;13(3):e002906. doi: 10.1136/bmjoq-2024-002906 (PMC11429364; doi:10.1136/bmjoq-2024-002906)
Supplement: online supplemental file 3 [file bmjoq-13-3-s003.pdf]

Appendix 3

Search Strategy for the Reviews

1. “Emergency Service, Hospital”[MeSH]
2. “Emergency Medical Services”[MeSH]
3. 1 OR 2
4. “Emergency Medicine”[MeSH]
5. 3 OR 4
6. "emergency service*"[TIAB] OR "emergency medical service*"[TIAB] OR "emergency care"[TIAB] OR "urgent care"[TIAB] OR "emergency department*"[TIAB] OR "accident and emergency"[TIAB] OR casualty[TIAB]

Filters: Meta-Analysis, Review, Systematic Review, English, from 2018 - 2024 Sort by: Publication Date

Google Scholar search

Free open source software Publish or Perish (https://harzing.com/resources/publish-or-perish/) was used to conduct systematic searches of Google Scholar. An alternative to arbitrary “scanning of the first X pages” this software allows construction of specific search strategies within a ceiling of 1000 results, storing both search strategy and results for further reference and download. Strategies included the following “keywords”: (i) (crowding OR overcrowding) AND "emergency department" AND "systematic review" [993 results]; "Systematic review" "hour wait" emergency [205 results]. Searches were conducted on the 11th and 12th January 2024

MEDLINE Search Strategies for Interventions where required (Tier 2 searches)

Review 1: Urgent Community Response

Ovid MEDLINE(R) Epub Ahead of Print and In-Process, In-Data-Review & Other Non-Indexed Citations <February 23, 2024>
1 "urgent community response".ab,ti. (1)
2 ucr.ab,ti. (850)
3 "rapid response service* ".ab,ti. (31)
4 hrrs.ab,ti. (289)
5 (community adj3 urgent care).ab,ti. (35)
6 "urgent home care".ab,ti. (1)
7 After-Hours Care/ (2116)
8 ("after hours" adj3 (service* or care or clinic* or centre* or center*)).ab,ti. (504)
9 ("out of hours" adj3 (service* or care or clinic* or center* or centre*)).ab,ti. (1148)
10 ((gp* or "general practice* or general practitioner*") adj3 ("out of hours" or co?operative* or collaborative*)).ab,ti. (479)
11 "on site paramedic* ".ab,ti. (3)
12 Access to Primary Care/ (35)
13 "access to primary care".ab,ti. (1539)
14 ("rural health" adj (clinic* or centre* or center*)).ab,ti. (1023)
15 1 or 2 or 3 or 4 or 5 or 6 or 7 or 8 or 9 or 10 or 11 or 12 or 13 or 14 (7046)
16 ((ED or emergency or A&E) adj5 (wait* or time* or duration or length or minute* or hour*)).ab,ti. (20750)

17 (ambulance* and ("response time*" or delay* or "waiting time*")).ab,ti. (1625)
18 16 or 17 (22076)
19 15 and 18 (434)
20 limit 19 to yr="2018 -Current" (139)

Review 2: Same Day Emergency Care

Ovid MEDLINE(R) and Epub Ahead of Print, In-Process, In-Data-Review & Other Non-Indexed Citations and Daily <1946 to February 12, 2024>

1	(same day emergency care or same-day emergency care).mp.	28
2	SDEC.ti,ab.	85
3	*Ambulatory Care/	20860
4	*Emergency Service, Hospital/	47543
5	*Emergency Medical Services/	36399
6	*Emergency Medicine/	11957
7	(emergency adj2 service*).ti,ab.	22063
8	"emergency care".ti,ab.	11778
9	"urgent care".ti,ab.	3363
10	"emergency department* ".ti,ab.	130816
11	"accident and emergency".ti,ab.	5049
12	casualty.ti,ab.	6807
13	or/4-12	210650
14	1 or 2	99
15	3 and 13	1284
16	ambulatory emergency care.ti,ab.	43
17	15 or 16	1316
18	AEC.ti,ab.	2625
19	13 and 18	30
20	17 or 19	1331
21	limit 20 to yr="2018 -Current"	403

Review 3: Acute Frailty

Ovid MEDLINE(R) ALL 1946 to February 27, 2024

#	Searches	Results
1	((frail* adj5 assessment) or (frail* adj5 urgent) or (acute adj5 frail*) or ("Older People's Assessment Liaison" or OPAL)).tw.	4627
2	*Emergency Service, Hospital/ or *Emergency Medical Services/ or *Emergency Medicine/	92746
3	((emergency adj2 service*) or "emergency care" or "urgent care" or "emergency department*" or "accident and emergency" or casualty).tw.	166244
4	1 and (2 or 3)	141
5	("frailty assessment unit*" or "frailty assessment and intervention*" or frailty-in-urgent-care or "frailty in urgent care" or "older people* assessment*" or "acute frailty unit*" or "acute frailty service*").tw.	42
6	4 or 5	179
7	limit 6 to (english language and yr="2018 -Current")	125

Review 4: In-patient flow

Review 5: Care Transfer Hubs

Review 6: Community Beds

Ovid MEDLINE(R) Epub Ahead of Print and In-Process, In-Data-Review & Other Non-Indexed Citations <February 22, 2024>

1	community beds.mp.	9
2	(step-down beds or step down beds or stepdown beds).mp.	18
3	(transitional care and beds).mp.	23
4	exp Transitional Care/	1313
5	exp Beds/ or exp Bed Occupancy/	7274
6	4 and 5	0
7	*Intermediate Care Facilities/	520
8	5 and 7	4
9	intermediate care beds.mp.	23
10	*Rehabilitation/	14655
11	*Rehabilitation Centers/	4307
12	10 or 11	18668
13	5 and 12	20
14	rehabilitation beds.mp.	56
15	(community recovery and beds).mp.	2
16	(local authorit* and beds).mp.	40
17	(NHS and maintain* and beds).mp.	13
18	((P2 or pathway 2) and beds).mp.	35
19	((D2A or "discharge to assess") and beds).mp.	2
20	((Pathway 2A or P2A) and beds).mp.	0
21	((block funded or block-funded or blockfunded) and beds).mp.	0
22	((spot purchased or spot-purchased) and beds).mp.	0
23	("any qualified provider framework" or AQP framework).mp.	1
24	"discharge to assess".mp.	203
25	community.mp.	744959
26	24 and 25	28
27	1 or 2 or 3 or 6 or 8 or 13 or 14 or 15 or 16 or 17 or 18 or 19 or 20 or 21 or 22 or 23 or 26	249
28	limit 27 to yr="2018 -Current"	74

Review 7: Intermediate Care

Ovid MEDLINE(R) ALL <1946 to February 14, 2024>

1	*Emergency Service, Hospital/	47539
2	*Emergency Medical Services/	36394
3	*Emergency Medicine/	11957
4	(emergency adj2 service*).ab,ti.	22064
5	"emergency care".ab,ti.	11778

6 "urgent care".ab,ti. 3368
7 "emergency department* ".ab,ti. 130878
8 "accident and emergency".ab,ti. 5047
9 casualty.ab,ti. 6808
10 exp *Critical Care/ 38577
11 *intensive care units/ or *recovery room/ 29500
12 or/1-11 272791
13 Intermediate Care Facilities/ or (intermediate care or IMCU or IMCUs).mp. 2379
14 (step-up or step-down or stepup or stepdown).mp. 7622
15 *subacute care/ 913
16 ((post-acute or sub-acute or subacute) adj3 care).ti. 852
17 *transitional care/ 1132
18 13 or 14 or 15 or 16 or 17 12442
19 12 and 18 790
20 *"continuity of patient care"/ or *patient transfer/ 16759
21 (outreach or out-reach or ((followup or follow* up) adj3 care)).mp. 31380
22 *home care services, hospital-based/ or *rehabilitation centers/ 5877
23 21 or 22 37194
24 20 and 23 673
25 12 and 24 113
26 19 or 25 899

Review 8: Single Point of Access

Ovid MEDLINE(R) and Epub Ahead of Print, In-Process, In-Data-Review & Other Non-Indexed Citations and Daily <1946 to February 08, 2024>

1 "single point of access".mp. 57
2 unified access point*.mp. 4
3 (front door access or front-door access).mp. 2
4 "centrali?ed access to care".mp. 1
5 single entry point*.mp. 69
6 "single point of triage".mp. 2
7 single triage point*.mp. 1
8 1 or 2 or 3 or 4 or 5 or 6 or 7 135
9 limit 8 to yr="2018 -Current" 52

Review 9: ARI hubs

Ovid MEDLINE(R) ALL <1946 to February 15, 2024>
1 ("respiratory clinical assessment service*" or "acute respiratory infection hub*" or "ARI hub*").mp. 5

2 (("Acute respiratory infection" adj3 hub*) or ("Acute respiratory tract infection" adj3 hub*) or ("respiratory tract infection" adj3 hub*) or ("Lower respiratory tract infection" adj3 hub*) or ("upper respiratory tract infection" adj3 hub*) or ("Acute lower respiratory tract infection" adj3 hub*) or ("Acute upper respiratory tract infection" adj3 hub*)).mp. 2

3 ("acute respiratory infection clinic" or "acute respiratory infection clinics").mp. 1

4 (("Acute respiratory infection" adj3 clinic) or ("Acute respiratory tract infection" adj3 clinic) or ("respiratory tract infection" adj3 clinic) or ("Lower respiratory tract infection" adj3 clinic) or ("upper respiratory tract infection" adj3 clinic) or ("Acute lower respiratory tract infection" adj3 clinic) or ("Acute upper respiratory tract infection" adj3 clinic)).mp. 17

5 (("Acute respiratory infection" adj3 clinics) or ("Acute respiratory tract infection" adj3 clinics) or ("respiratory tract infection" adj3 clinics) or ("Lower respiratory tract infection" adj3 clinics) or ("upper respiratory tract infection" adj3 clinics) or ("Acute lower respiratory tract infection" adj3 clinics) or ("Acute upper respiratory tract infection" adj3 clinics)).mp. 1

6 ("acute respiratory infection centre*" or "acute respiratory infection center*").mp. 0

7 (("Acute respiratory infection" adj3 centre*) or ("Acute respiratory tract infection" adj3 centre*) or ("respiratory tract infection" adj3 centre*) or ("Lower respiratory tract infection" adj3 centre*) or ("upper respiratory tract infection" adj3 centre*) or ("Acute lower respiratory tract infection" adj3 centre*) or ("Acute upper respiratory tract infection" adj3 centre*)).mp. 4

8 (("Acute respiratory infection" adj3 center*) or ("Acute respiratory tract infection" adj3 center*) or ("respiratory tract infection" adj3 center*) or ("Lower respiratory tract infection" adj3 center*) or ("upper respiratory tract infection" adj3 center*) or ("Acute lower respiratory tract infection" adj3 center*) or ("Acute upper respiratory tract infection" adj3 center*)).mp. 15

9 ("Acute respiratory infection care clinic" or "Acute respiratory tract infection care clinic" or "respiratory tract infection care clinic" or "Lower respiratory tract infection care clinic" or "upper respiratory tract infection care clinic" or "Acute lower respiratory tract infection care clinic" or "Acute upper respiratory tract infection care clinic").mp.0

10 ("Acute respiratory infection care clinics" or "Acute respiratory tract infection care clinics" or "respiratory tract infection care clinics" or "Lower respiratory tract infection care clinics" or "upper respiratory tract infection care clinics" or "Acute lower respiratory tract infection care clinics" or "Acute upper respiratory tract infection care clinics").mp. 0

11 ("Acute respiratory infection assessment centre*" or "Acute respiratory tract infection assessment centre*" or "respiratory tract infection assessment centre*" or "Lower respiratory tract infection assessment centre*" or "upper respiratory tract infection assessment centre*" or "Acute lower respiratory tract infection assessment centre*" or "Acute upper respiratory tract infection assessment centre*").mp. 0

12 ("Acute respiratory infection assessment center*" or "Acute respiratory tract infection assessment center*" or "respiratory tract infection assessment center*" or "Lower respiratory tract infection assessment center*" or "upper respiratory tract infection assessment center*" or "Acute lower respiratory tract infection assessment center*" or "Acute upper respiratory tract infection assessment center*").mp. 0

13 ("Acute respiratory infection treatment centre*" or "Acute respiratory tract infection treatment centre*" or "respiratory tract infection treatment centre*" or "Lower respiratory tract infection treatment centre*" or "upper respiratory tract infection treatment centre*" or "Acute lower respiratory tract infection treatment centre*" or "Acute upper respiratory tract infection treatment centre*").mp. 0

14 ("Acute respiratory infection treatment center*" or "Acute respiratory tract infection treatment center*" or "respiratory tract infection treatment center*" or "Lower respiratory tract infection treatment center*" or "upper respiratory tract infection treatment center*" or "Acute lower respiratory tract infection treatment center*" or "Acute upper respiratory tract infection treatment center*").mp. 0

15 ("Acute respiratory infection clinical assessment service*" or "Acute respiratory tract infection clinical assessment service*" or "respiratory tract infection clinical assessment service*" or "Lower respiratory tract infection clinical assessment service*" or "upper respiratory tract infection clinical assessment service*" or "Acute lower respiratory tract infection clinical assessment service*" or "Acute upper respiratory tract infection clinical assessment service*").mp. 0

16 ((respiratory adj2 "care clinic") or (respiratory adj2 "care clinics") or (respiratory adj2 "assessment centre*") or (respiratory adj2 "assessment center*") or (respiratory adj2 "treatment

centre*") or (respiratory adj2 "treatment center*") or (respiratory adj2 "clinical assessment service*")).mp. 8

17 ("ARI clinic" or "ARI clinics" or "ARI centre*" or "ARI center*" or "ARI care clinic" or "ARI care clinics" or "ARI assessment centre*" or " ARI assessment center*" or "ARI treatment centre*" or "ARI treatment center*" or "ARI clinical assessment service*").mp. 2

18 ("ARTI hub*" or "ARTI clinic" or "ARTI clinics" or "ARTI centre*" or "ARTI center*" or "ARTI care clinic" or "ARTI care clinics" or "ARTI assessment centre*" or " ARTI assessment center*" or "ARTI treatment centre*" or "ARTI treatment center*" or "ARTI clinical assessment service*").mp. 0

19 ("RTI hub*" or "RTI clinic" or "RTI clinics" or "RTI centre*" or "RTI center*" or "RTI care clinic" or "RTI care clinics" or "RTI assessment centre*" or " RTI assessment center*" or "RTI treatment centre*" or "RTI treatment center*" or "RTI clinical assessment service*").mp. 8

20 ("LRTI hub*" or "LRTI clinic" or "LRTI clinics" or "LRTI centre*" or "LRTI center*" or "LRTI care clinic" or "LRTI care clinics" or "LRTI assessment centre*" or " LRTI assessment center*" or "LRTI treatment centre*" or "LRTI treatment center*" or "LRTI clinical assessment service*").mp. 0

21 ("URTI hub*" or "URTI clinic" or "URTI clinics" or "URTI centre*" or "URTI center*" or "URTI care clinic" or "URTI care clinics" or "URTI assessment centre*" or " URTI assessment center*" or "URTI treatment centre*" or "URTI treatment center*" or "URTI clinical assessment service*").mp. 1

22 ("ALRI hub*" or "ALRI clinic" or "ALRI clinics" or "ALRI centre*" or "ALRI center*" or "ALRI care clinic" or "ALRI care clinics" or "ALRI assessment centre*" or " ALRI assessment center*" or "ALRI treatment centre*" or "ALRI treatment center*" or "ALRI clinical assessment service*").mp. 1

23 ("AURI hub*" or "AURI clinic" or "AURI clinics" or "AURI centre*" or "AURI center*" or "AURI care clinic" or "AURI care clinics" or "AURI assessment centre*" or " AURI assessment center*" or "AURI treatment centre*" or "AURI treatment center*" or "AURI clinical assessment service*").mp. 0

24 1 or 2 or 3 or 4 or 5 or 6 or 7 or 8 or 9 or 10 or 11 or 12 or 13 or 14 or 15 or 16 or 17 or 18 or 19 or 20 or 21 or 22 or 23 60

25 limit 24 to yr="2018 -Current" 30

19 ("RTI hub*" or "RTI clinic" or "RTI clinics" or "RTI centre*" or "RTI center*" or "RTI care clinic" or "RTI care clinics" or "RTI assessment centre*" or " RTI assessment center*" or "RTI treatment centre*" or "RTI treatment center*" or "RTI clinical assessment service*").mp. 11

20 ("LRTI hub*" or "LRTI clinic" or "LRTI clinics" or "LRTI centre*" or "LRTI center*" or "LRTI care clinic" or "LRTI care clinics" or "LRTI assessment centre*" or " LRTI assessment center*" or "LRTI treatment centre*" or "LRTI treatment center*" or "LRTI clinical assessment service*").mp. 0

21 ("URTI hub*" or "URTI clinic" or "URTI clinics" or "URTI centre*" or "URTI center*" or "URTI care clinic" or "URTI care clinics" or "URTI assessment centre*" or " URTI assessment center*" or "URTI treatment centre*" or "URTI treatment center*" or "URTI clinical assessment service*").mp. 0

22 ("ALRI hub*" or "ALRI clinic" or "ALRI clinics" or "ALRI centre*" or "ALRI center*" or "ALRI care clinic" or "ALRI care clinics" or "ALRI assessment centre*" or " ALRI assessment center*" or "ALRI treatment centre*" or "ALRI treatment center*" or "ALRI clinical assessment service*").mp. 0

23 ("AURI hub*" or "AURI clinic" or "AURI clinics" or "AURI centre*" or "AURI center*" or "AURI care clinic" or "AURI care clinics" or "AURI assessment centre*" or " AURI assessment center*" or "AURI treatment centre*" or "AURI treatment center*" or "AURI clinical assessment service*").mp. 0

24 1 or 2 or 3 or 4 or 5 or 6 or 7 or 8 or 9 or 10 or 11 or 12 or 13 or 14 or 15 or 16 or 17 or 18 or 19 or 20 or 21 or 22 or 23 80

25 limit 24 to yr="2018 -Current" 34

Review 10: Virtual Wards

Ovid MEDLINE(R) ALL <1946 to February 26, 2024>
1 ("virtual ward*" or (virtual* adj3 ward*)).tw. 133
2 ("hospital? at home" or hah).tw. 1068
3 "hospital? in the home".tw. 223
4 ("home?based hospital?" or (hospital? adj3 home?based)).tw. 1
5 ("home hospitali#ation?" or (hospitali#ed adj3 home)).tw. 586
6 (home?based care or "advanced care at home").tw. 27
7 (remote* adj2 patient monitor*).tw. 790
8 1 or 2 or 4 or 5 or 6 or 7 2534
9 *Emergency Service, Hospital/ 47565
10 *Emergency Medical Services/ 36464
11 *Emergency Medicine/ 11968
12 (emergency adj2 service*).tw. 22117
13 "emergency care".tw. 11807
14 "urgent care".tw. 3372
15 "emergency department* ".tw. 131295
16 "accident and emergency".tw. 5051
17 casualty.tw. 6823
18 9 or 10 or 11 or 12 or 13 or 14 or 15 or 16 or 17 211241
19 8 and 18 192
20 limit 19 to yr="2018 -Current" 133
