# Supplementary material for: Scoping review of the effectiveness of 10 high-impact initiatives (HIIs) for recovering urgent and emergency care services
Source: BMJ Open Qual. 2024 Sep 18;13(3):e002906. doi: 10.1136/bmjoq-2024-002906 (PMC11429364; doi:10.1136/bmjoq-2024-002906)
Supplement: online supplemental file 4 [file bmjoq-13-3-s004.pdf]

Appendix 4: Summary of Findings and Implications

High Impact Initiative	Quantity and Study Types of Evidence Base	Brief Summary of Findings	Overall Conclusion	Implications for Decision Makers
**Urgent Community Response (UCR)**	Tier 1 - n=5<br><br>Relevant studies in any review (range 1-3); studies from Sweden and UK.<br><br>Tier 2: n=0	UCR serves to treat patients at home or in the community to avoid ED admission. Includes extended care paramedics within long-term care settings, mobile integrated health community paramedicine programs, improved access to primary care (e.g., out of hours and after hours care). Relevant findings from a small number of Tier 2 studies (n=5) reported in Tier 1 - Review level reviews (n=5). Limited evidence that such interventions may reduce ambulance mission and duty times, as well as ED wait times.	Very limited international evidence to suggest that UCR is effective in terms of reduced ambulance response times and ED wait times.<br><br>However, this may be because these metrics are not usually reported in studies evaluating the impact of such interventions on health services.	**Urgent Community Response** covers a set of very different interventions. It is challenging to conclude that any one particular intervention is effective in improving ambulance response times or ED wait times.<br><br>**Given that Urgent Community Response has typically been measured using alternative measures the link between the interventions and the outcomes of interest needs to be more positively established.**
**Same Day Emergency Care (SDEC) / Ambulatory Emergency Care (AEC)**	Tier 1: n=1<br><br>1 review (reporting ED wait times) (only included two of the Tier 2 studies reported below); studies from UK.<br><br>Tier 2: n=6<br><br>1 cross-sectional comparative study, 1 prospective cohort study, 1 observational study and audit; 3 retrospective audits, all from the UK	Relevant findings for ED wait times from a small number of UK Tier 2 studies (n=6). All studies found reduced ED wait times.<br><br>No evidence on ambulance response times.	Limited but consistent UK evidence to suggest that SDEC/AEC is effective in terms of reduced ED wait times.<br><br>No evidence on ambulance response times.	**Same Day Emergency Care** services are a clearly-defined type of service. They are supported by a limited number of primary research studies. Only one review exists.<br><br>**Given that limited but consistent highly relevant evidence exists, SDEC services could be continued within a**

High Impact Initiative	Quantity and Study Types of Evidence Base	Brief Summary of Findings	Overall Conclusion	Implications for Decision Makers
				**context of ongioing rigorous evaluation.**
**Acute Frailty**	Tier 1: n=3<br><br>Relevant studies in any review (range 1-6); studies from UK, Belgium, Finland, Italy, Switzerland, USA, Australia,<br><br>Tier 2: n=3<br><br>1 RCT, 1 prospective observational studies, 1 pilot evaluation; studies from UK, Finland	Variation persists in how frailty is defined, and the services evaluated relate to different approaches to frailty assessment and different staffing models of care. Evidence base primarily relates to ED or ICU length of stay as a proxy for wait time.<br><br>Relevant findings for ED wait times from a moderate number of studies across Tiers 1 and 2. Overall, the evidence at all Tiers indicates a reduction in ED wait time or a trend towards shorter wait times.<br><br>No evidence on ambulance response times.	Moderate international evidence to suggest that versions of acute frailty services within or related to the ED might be effective in terms of reducing ED wait times.<br><br>No evidence on ambulance response times.	Approaches to **Acute Frailty** are very varied, both nationally and internationally. Research on acute frailty often extends beyond the emergency department to length of stay in hospital or in an ICU.<br><br>**Given that approaches to acute frailty extend from the community through the ED to other parts of the hospital,l research should study impact along the whole acute frailty pathway.**
**In-Patient Flow**	Tier 1 - Review level: n=33<br><br>Relevant studies in these review (n=>200); studies from an international evidence base.<br><br>Tiers 2: Not applicable.	The included interventions were broadly categorised as Teams-based, Roles, Pathways or specific patient groups, Units, Technology, Bed management, Point of care testing, Target-based, Protocols and standardisation of processes, and Other.<br><br>Relevant findings for ED wait times from Tier 1 studies (n=33). Under each category, generally effects are inconclusive: a majority might report finding in favour of	Strong/moderate international evidence to suggest that some types of in-patient flow interventions within or related to the ED might be effective in terms of reducing ED wait times.<br><br>Weak international evidence to suggest that some types of in-patient flow interventions within or related to the ED might be effective in	In-patient flow typically extends to between 30 and 40 different interventions. This heterogeneity provides a challenge to evaluation but ED wait times and lenth of stay are commonly measured.<br><br>**Given that in-patient flow is well studied across multiple diverse intervention types more attention could be paid on establishing which approaches**

High Impact Initiative	Quantity and Study Types of Evidence Base	Brief Summary of Findings	Overall Conclusion	Implications for Decision Makers
		interventions, but some reviews do report finding no difference between interventions and previous practice, and some an increase in ED wait times with the intervention.<br><br>Many studies evaluate reduction in persons leaving without being seen.<br><br>No evidence on ambulance response times, only ambulance offload delay (Tier 1 - Review level, n=1, from 14 Tier 2/3 studies). Evidence was principally related to ambulance diversion.	terms of reducing ambulance offload delay.<br><br>However, it is challenging to evaluate individual interventions. Often In-Patient Flow interventions are part of a bundle (opportunistic interventions) or Quality Improvement (QI) package (planned multi-intervention).	**are most effective. Evidence suggests that targeting in-patient flow with a designated member of staff may achieve a small but meaningful effect, at least in the short-term.**
**Care transfer hubs**	Tier 1: n=2<br><br>Relevant studies in these reviews (n=5); studies from UK, USA, Belgium, Australia.<br><br>Tier 2: Not applicable.	The Tier 1 evidence evaluated the impact of a range of interventions labelled as transitional care interventions (TCIs) implemented within the ED.<br><br>Relevant findings for ED wait times from a small number of Tier 3 studies (n=5) reported in the Tier 1 reviews (n=2). One review reported a clear reduction in ED wait times for a multidiisciplinary team led intervention, but the second review of a nurse-led intervention reported more inconclusive findings.	Weak international evidence to suggest that Care Transfer Hubs are effective in terms of reduced ED wait times.<br><br>However, it is challenging to define Care Transfer Hubs within the literature. Studies tend to include one or more components of Care Transfer hubs but rarely include all components or identify intervention as a "hub".<br><br>No evidence on ambulance response times.	Care Transfer Hubs are insufficiently distinguished with the research agenda and are typically evaluated through their components rather than as discrete entities.<br><br>**Given a lack of clarity around what services such hubs provide commissioned research around an agreed package of services (compare research evaluations of GPs in Emergency Departments) may help to address knowledge deficits.**

**High Impact Initiative**	**Quantity and Study Types of Evidence Base**	**Brief Summary of Findings**	**Overall Conclusion**	**Implications for Decision Makers**
		No evidence on ambulance response times.		
**Community beds**	Tier 1 n=0<br>Tier 2: n=0	No relevant findings for ED wait times or ambulance response times, or similar ED time-related outcomes. No relevant studies were identified.	Research with higher-quality design needed to examine impact of community beds on ED outcomes, including wait times and ambulance response times.	**Community beds** are insufficiently evaluated, perhaps given the absence of leadership of a potential research agenda.<br>**Given the dearth of research greater utilisation of routine data sources may well be a preferred approach to short-term evaluation attempts.**
**Intermediate care interventions**	Tier 1: n=3<br>Relevant studies in these reviews (n=unclear); studies from an international evidence base.<br>Tier 2: Not applicable.	Relevant findings for ED wait times from Tier 1 studies (n=3). Specific intermediate care interventions (e.g., Transitional Care Interventions, Virtual Wards, tele-monitoring) were found to be associated with shorter LOS and waiting times in ED.<br>No evidence on ambulance response times.	Moderate international evidence to suggest intermediate care interventions might be effective in reducing ED-LOS.<br>No evidence on ambulance response times.<br>Further research needed to identify most effective intervention components and how hybrid intervention approaches (pre- to post-discharge) can be smoothly implemented with reference to particular high-risk patient groups.	**Intermediate care approaches** take multiple diverse forms. Diverse forms evaluate differently.<br>**Given the heterogeneity of intermediate care approaches, recommendation of interventions could focus on specific named interventions rather than intermediate care more generally.**
**Single Point of Access (SPoA)**	Tier 1: n=7.<br>7 systematic reviews (international evidence base) on role of GP or	Interventions generally evaluated role of General Practitioners or advanced practice nurses in non-	Moderate international evidence suggesting that integrating primary care services or providers within or	**Single point of access** is poorly defined. It is commonly understood as involving services

High Impact Initiative	Quantity and Study Types of Evidence Base	Brief Summary of Findings	Overall Conclusion	Implications for Decision Makers
	other primary practitioners within or alongside ED examining ED Wait Times. One review (shared with #1) on ambulance outcomes (duty cycle time and conveyance rates, with 21 relevant studies from Norway, UK, Sweden, Belgium, Switzerland and New Zealand).<br><br>Tier 2: n=6.<br><br>Observational and/or cross-sectional studies, 1 mixed-method study; studies focused on models of GP co-location or co-operation; studies from the UK, Netherlands, Germany and Australia.	critical emergency medicine settings (both within or alongside the ED, but not in the community).<br><br>Relevant findings from Tier 1 and Tier 2: Major UK GPED study (multiple reports (n =2) but little data). Ability to conclude from GPED case studies (n = 10) limited by different interpretation of models and contextually-sensitive differences. GPs also perceive that they are working harder.<br><br>Relevant findings from Tier 1: one review (21 relevant international studies) found that having a SPoA reduced ambulance duty cycles and conveyance rates.	alongside EDs can potentially reduce ED LOS for certain patient groups, but the impacts are not consistent across all settings and depend on various factors related to service design and implementation.<br><br>Some evidence from Northern Europe that greater availability may lead to improved access and greater demand. Not confirmed by UK GPED study.<br><br>Weak international evidence suggesting SPoAs might have a positive effect on some ambulance time-related metrics.	such as NHS111 but the recent research agenda has focused on GPs and advanced nursing staff within or alongside the emergency department.<br><br>**Given the mismatch between SPoA as a concept and the very specific evaluation of GP and advanced nursing roles NHS England could decide whether to recommend more specific interventions. However, success of such interventions is context-sensitive. Realtively strong evidence exists for the impact of short-stay crisis units on ED-LOS.**
**Acute Respiratory Infection (ARI) hubs**	Tier 1: n=2:<br><br>Relevant studies in these reviews (n=9); studies from UK, USA and Canada. One review of adults with acute respiratory distress, one in children and adolescents.<br><br>Tier 2: n=1<br><br>One prospective, longitudinal, participatory action research study from UK.	Relevant findings for ED wait times from Tier 1: Modified asthma pathways delivered by nurse-led teams effective in reducing time to treatment (ED-LOS). Individual caseworker-assigned plans assigned to children and adolescents with AREs not effective to reduce likelihood of subsequent ED visits.<br><br>Findings for ED wait times from Tier 3: Reduction in ED visits.	Very weak evidence to suggest ARI hubs might reduce ED wait times. Scarcity of data does not permit robust conclusions for ARI hub effects on healthcare utilisation and patient-related outcomes.<br><br>No evidence on ambulance response times.<br><br>Recently published NICE guideline [NICE guideline NG237] did not identify any studies providing	**Given the significant lack of research studies, particularly outside a pandemic environment, further recommendation of ARIs as an intervention should be predicated on robust evaluation and research evidence.**

**High Impact Initiative**	**Quantity and Study Types of Evidence Base**	**Brief Summary of Findings**	**Overall Conclusion**	**Implications for Decision Makers**
		No evidence on ambulance response times.	evidence regarding effectiveness of ARI hubs (p.65).	
**Virtual Wards / Hospital at Home (HAH)**	Tier 1: n=3.<br><br>Relevant studies in these reviews (n=18); studies evaluating prehospital telemedicine were from an international evidence base: UK, USA, Denmark, Germany, Hong Kong, Spain, Italy, Turkey, USA. Canada.<br><br>Tier 2: n=1<br><br>One non-randomised prospective case–controlled study evaluating HAH from the USA	Relevant findings for ED wait times and ambulance response from Tier 1: One review of prehospital telepsychiatry (with 1 relevant USA study) reported reduction in ED wait times. Three reviews found either a reduction in ambulance response times (1 review with 3 relevant studies) or a reduction in 'time to treatment' (2 reviews with 15 relevant studies) for interventions involving prehospital telemedicine.<br><br>Relevant findings for ED wait times from Tier 2: 1 primary research study specifically evaluated an HAH intervention and found a reduction in ED wait times.	Weak evidence to suggest prehospital telemedicine or HAH might reduce ED wait times.<br><br>Moderate international evidence suggests prehospital telemedicine might reduce ambulance response times or time to treatment in some groups.	Drivers for telemedicine and **virtual wards/hospital at home** focus more on convenience and cost reduction.<br><br>**Given weak overall evidence in favour of virtual wards and hospital at home interventions in terms of the headline metrics such interventions could be presented as broadening access and expanding the urgent and emergency care service portfolio.**

ALC: Alternate level of care; ARE: Acute respiratory exacerbation; ARI: Acute response infection; ED: Emergency department; LOS: Length of stay; MA: Meta-analysis; SAFE: Sub-Acute care for Frail Elderly; SR: Systematic review
