# Supplementary material for: Scoping review of the effectiveness of 10 high-impact initiatives (HIIs) for recovering urgent and emergency care services
Source: BMJ Open Qual. 2024 Sep 18;13(3):e002906. doi: 10.1136/bmjoq-2024-002906 (PMC11429364; doi:10.1136/bmjoq-2024-002906)
Supplement: online supplemental file 5 [file bmjoq-13-3-s005.pdf]

Appendix 5: Summary tables for each review

1. Urgent Community Response (UCR):

Table S1.1: ED waiting time-based metrics (Tier 1)

First author year (ref)	Country & sample size (n=studies)	Study design	Aim	Intervention<br><br>Population	Findings<br><br>Other ED outcomes reported
Doshmangir 2022	Total, n=64<br><br>Australia(4), Bahrain(1), Canada(4), China(1), Korea(1), Netherlands(1), Singapore(1), Sweden(3), Tawain(1), UK(3), USA(34)<br><br>Relevant studies, n=1<br>Canada(1)	Systematic review	Investigating initiatives intended to manage adult hospital services and reduce unnecessary hospital use among the general adult population	Advanced life support drug treatment given by ambulance attendants (in community)	Wait times: none<br><br>ED-LOS:<br>Before = 206.9 min; After = 220.9 min; Difference = +14 min (not statistically significant) (Ferrazzi et al, 2001). Canada. Overall quality assessment rating was "weak".<br><br>Other outcomes: proportion of admissions
Jeyaraman 2021	Total, n=268,<br>North America (156), Europe (76), Oceana (22), Asia (9), Middle East (3), South America (1), NR 1)<br><br>Relevant studies, n=1 (unclear)	Scoping review	To identify and summarise the existing literature on interventions involving primary healthcare professionals to manage emergency department (ED) overcrowding	Urgent care collaborations between the GP and ED (includes patients presenting GP)<br>Patients with asthma/ chronic obstructive pulmonary disease (COPD) or a sprained ankle presenting to the ED and/or GP	Wait times:<br>Patients seeking ED care due to lack of timely primary care access (same-day or after-hours appointments) may contribute to ED overcrowding and in this context some of the 'outside ED' interventions summarised in this scoping review may be beneficial in mitigating ED overcrowding. Van Gils-van Rooij (2018) reported a negative effect on wait times.<br><br>Other ED outcomes: ED visits, Number of patients diverted to Primary Care, Patient safety, Patient satisfaction, Total cost, Volume of patients visiting ED
Leduc 2020	Total, n=22<br>Canada(5), Norway(1), Scotland1), USA(15)<br><br>Relevant, n=1 (country not reported)	Systematic review	To determine, among long-term care patients, what is the effectiveness and safety of interventions that evaluate and treat patients on site, avoiding unscheduled transport to the ED	Extended Care Paramedics (ECP) to respond to acute illnesses in residents<br><br>Long-term care residents	Wait times:<br>When transported to hospital, patients spent less time in the ED before being admitted or discharged if they were in the ECP group compared to those treated by emergency paramedics. [Jensen 2016] Risk of bias for as assessed by the Newcastle-Ottawa scale: 7 out of 9.<br><br>Other ED outcomes: ED transports, hospital admissions, cost savings, patient-specific outcomes, mortality, rates of relapse

Pearce 2023	Total, n = 27<br><br>Included, n = 3	Overview of reviews	To summarize the interventions at each level of emergency care: input, throughput, and output	After-hours access to primary care	Results were mixed but overall reduced likelihood of ED utilization. In some cases, it reduced composite metrics of crowding and length of stay [Morley 2018; Hong 2020; Rasouli 2019]<br><br>Interventions reported as having moderate sustained results.<br><br>Other ED outcomes: ED visits, Savings

Table S1.2: Ambulance time-based metrics (Tier 1)

First author year (ref)	Country & sample size (n=studies)	Study design	Aim	Intervention<br><br>Population	Findings<br><br>Other ED outcomes reported
Burrell 2023	Total, n=21, Belgium(1), Norway(11), NZ(1), Sweden(3), Switzerland(1), UK(4)<br><br>Relevant studies, n=2 Sweden(1), UK(1)	Mapping review	To collate and summarise evidence on how GPs are utilised in EMS	Referral to GP by ambulance staff<br><br>GP Acute Visiting Scheme (AVS)<br><br>General	**Ambulance mission time:** In the Swedish study, patients referred to a GP by the EMS triaging nurse [had] a significantly shorter mean ambulance mission time (86.88 versus 94.12 minutes, P= 0.04).[Larsson 2017]. Risk of bias: key (NB This was the highest possible quality rating).<br><br>**Ambulance duty cycle time:** Ambulance duty cycle time for a GP AVS referral was, on average, 15 minutes less than for missions resulting in direct ED conveyance.[Blodgett 2017]. Risk of bias: Satisfactory (preliminary results, descriptive stats only) in terms of quality, but very relevant.<br><br>**Other ED outcomes:** ED admission avoidance, non-conveyance to ED

Doshmangir 2022	Total, n=64<br>Australia(4), Bahrain(1), Canada(4), China(1), Korea(1), Netherlands(1), Singapore(1), Sweden(3), Tawain(1), UK(3), USA(34)<br><br>Relevant studies, n=1<br>Canada(1)	Systematic review	Investigating initiatives intended to manage adult hospital services and reduce unnecessary hospital use among the general adult population	Advanced life support drug treatment given by ambulance attendants (in community)	Ambulance scene time:<br>Before = 12.3 min; After = 14.2 min (statistically significant) (Ferrazzi et al, 2001). Canada. Overall quality assessment rating was "weak".<br><br>Other outcomes: ED-LOS and proportion of admissions
Leduc 2020	Total, n=22<br>Canada(5), Norway(1), Scotland1), USA(15)<br><br>Relevant, n=1 (country not reported)	Systematic review	To determine, among long-term care patients, what is the effectiveness and safety of interventions that evaluate and treat patients on site, avoiding unscheduled transport to the ED	Extended Care Paramedics (ECP) to respond to acute illnesses in residents<br><br>Long-term care residents	Ambulance offload delay:<br>The study measuring out-of-hospital impacts reported there was no difference in offload delay between the intervention (Extended Care Paramedic) and control group (Emergency Paramedic). [Jensen 2016]<br>Risk of bias for as assessed by the Newcastle-Ottawa scale: 7 out of 9.<br>Other ED outcomes: ED transports, hospital admissions, cost savings, patient-specific outcomes, mortality, rates of relapse

References:

Burrell A, Scrimgeour G, Booker M. GP roles in emergency medical services: a systematic mapping review and narrative synthesis. *BJGP Open.* 2023;7(2).

Doshmangir L, Khabiri R, Jabbari H, Arab-Zozani M, Kakemam E, Gordeev VS. Strategies for utilisation management of hospital services: a systematic review of interventions. *Global Health.* 2022;18(1):53.

Jeyaraman MM, Copstein L, Al-Yousif N, et al. Interventions and strategies involving primary healthcare professionals to manage emergency department overcrowding: a scoping review. BMJ Open. 2021;11(5):e048613.

Leduc S, Cantor Z, Kelly P, Thiruganasambandamoorthy V, Wells G, Vaillancourt C. The Safety and Effectiveness of On-Site Paramedic and Allied Health Treatment Interventions Targeting the Reduction of Emergency Department Visits by Long-Term Care Patients: Systematic Review. Prehosp Emerg Care. 2021;25(4):556-565.

Pearce, S., Marr, E., Shannon, T., Marchand, T., & Lang, E. (2023). Overcrowding in emergency departments: an overview of reviews describing global solutions and their outcomes. *Internal and Emergency Medicine*, 1-9.

2. Same Day Emergency Care (SDEC):

Table S2.1: ED waiting time-based metrics (Tier 1)

First author year (ref)	Country & sample size (n=studies)	Study design	Aim	Intervention<br><br>Population	Findings<br><br>Other ED outcomes reported
Dean & Barratt 2024	Total, n=6<br><br>UK (6)<br><br>Relevant studies, n=2<br>UK (2)	Scoping review	To determine the existing evidence in relation to UK-based SDEC	SDEC<br><br>General	Wait times:<br>Average waiting time for ED 5 hours 44 minutes, compared to 49 minutes in SDEC (Jarral et al, 2020)<br><br>Reduction in time to senior medical review from 3 hours 19 minutes to 2 hours 56 minutes<br>Reduction in decision to discharge time from 3 hours 28 minutes to 2 hours 30 minutes (Visanji et al, 2020)<br><br>Other outcomes: Admission avoidance, safety, pressure on ED

Table S2.1.1: ED waiting time-based metrics (Tier 2)

First author year (ref)	Country & sample size (n=studies)	Study design	Aim	Methods & Outcomes	Intervention<br><br>Population	Findings<br><br>Other ED outcomes reported
Ali & Karami, 2018	UK (England)<br><br>Single centre<br><br>n=260 attendances	Audit (Abstract only)	To look at the performance of newly-developed ambulatory care service	Ambulatory care patients were selected who attended the service from 1st of October 2017 to 30th of October 2017.<br><br>Information was collected by looking at the ambulatory care record spreadsheet, medical notes, discharge letters and hospital ambulatory care data.<br><br>Service impact was analysed by numbers of service consumers, source of referral, impact on Accident and Emergency four hours target, appropriateness of referral, zero day discharges, length of stay for inpatients and diagnosis at discharge	AEC: Patients were admitted to a newly developed ambulatory care service using the AMBS criteria and different ambulatory care models over a 30-day period<br><br>General	Improvement in Accident and Emergency four hour target exceeded to 5%<br><br>**Other ED outcomes:** Referrals, discharge, inpatient LoS
Atkin et al, 2023	UK<br>Multi-centre<br>n=152 units in 149 UK hospitals	Cross-sectional comparative study	To evaluate performance of acute medicine services, assessing	152 acute UK hospital services accepting unplanned admissions to acute and general internal medicine completed a day-of-care survey incorporating organisational structure questionnaire and patient-level data over a pre-defined 24-hour period in June 2022.	SDEC vs Acute Medical Units (AMU) vs ED for unplanned admissions to acute and general internal medicine.	SDEC data only:<br>Those receiving initial assessment directly in SDEC services were more likely to have initial clinician

	7293 sequential patients were included in 2022 (and compared with 19,817 patients across 2019–2021)		clinical quality indicators for unplanned medical admissions to acute hospital services	All medical attendances arriving to hospital within a 24-hour period (00:00–23:59) were included, with data for collected for each patient describing demographic variables and processes of care.<br><br>Performance against clinical quality indicators was performed between SAMBA data collection periods in 2019, 2020 and 2021. As in 2022, data collection in 2019 and 2021 took place on the penultimate Thursday in June. Data collection in 2020 took place over 24 h on 30th January 2020. This date was chosen to be 6 months from June data collection rounds. Data collection did not take place in June 2020 due to pressures related to Covid-19; no rounds of SAMBA data collection have taken place during peaks of Covid-19 infection<br><br>Clinical quality indicators were: Early Warning Score (EWS) measurement within 30 min of hospital arrival; clinician assessment within 4 hours; assessment by consultant physician within 6 hours (daytime) or 14 hours (night-time). Results were compared with 2019, 2020, 2021.	Those expected to be discharged without overnight admission are assessed through SDEC services, receiving assessment, investigations, and management without admission to an inpatient bed. AMUs are the recommended care model for unplanned medical admissions in the UK; patients can remain on AMUs for the first 48–72 h of their admission, transferred to inpatient wards if requiring admission beyond 48 h.<br><br>General	assessment within 4 hours compared to those seen in AMU or ED (OR 2.4, 95%CI 2.02–2.87, *p*<0.001)....<br><br>Those initially assessed in AMU were more likely to see the medical team within 4 hours than those attending via ED (76.2% vs 34.4%, OR 6.1, 95%CI 5.03–7.44, *p*<0.001); as were those assessed directly in SDEC (87.6%, OR 13.4, 95%CI 11.4–15.9, *p*<0.001).<br><br>**Other ED outcomes:** Consultant review within 6 hours and 14 hours; Medical team assessment, admission to ICU, mortality
Edison et al, 2021	UK (England) Single centre n=175 patients	Prospective cohort study	To evaluate clinical outcomes of an emergency surgery AEC pathway implemented by combining a Physician associate (PA) with a senior decision-maker	An emergency surgery AEC pathway was introduced to a single centre in anticipation of a second COVID-19 wave. All emergency surgical referrals were prospectively collected over 3 months (November 2020 to February 2021) with minimum 30-day follow-up. Patients were referred to AEC following first attendance at Accident & Emergency (A&E) Department, Urgent Care Centre (UCC) or their general practitioner (GP), or as part of early facilitated discharge (EFD) from an inpatient admission. Referrals were made in real time using an electronic referral form, filled in by the relevant clinician via the trust's electronic healthcare records (EHR) platform.<br><br>Median time from request for senior review to senior treatment decision	Emergency general surgery AEC pathway staffed by new AEC physician's associate (PA) role supported by senior clinical decision-makers over 3-month period<br><br>Clinical presentations appropriate for AEC referral were categorised by types of pain, abscess, wound and 'other'	The median time from request for senior review to treatment decision was 26 min (IQR 9–62 min).<br><br>**Other ED outcomes:** Discharge rate

Jarral et al, 2020	UK (England)<br>Single centre<br>n=33 patients were taken directly from ED streaming	Audit (Abstract only)	To assess a pathway of direct referrals from ED streaming to ambulatory care	4-day period in September 2019<br>Patients were identified from ED streaming with chest pain, shortness of breath (SOB), palpitations and headache and fast tracked to ambulatory care. Exclusion criteria were used to ensure safety	Direct referral pathway from ED streaming to ambulatory care for clinically suitable patients with common conditions like chest pain, palpitations, headache or Shortness of breath/ chest infection.	Average waiting time for ED during week of pilot was 5 hours 44 minutes as compared with 49 minutes in ambulatory care during the same time period
Visanji et al, 2020	UK (England)<br>Single centre<br><br>n=NR	Prospective audit (Abstract only)	To look at the performance of a new acute medical unit (AMU), which uses the objectives and aims set out by NHS Improvement 'same-day emergency care' (SDEC).	Data were collected on time to triage, first medical review, senior decision maker and decision to discharge.<br><br>Plan, do, study, act cycles were used throughout the project and data collected prospectively from January 2019	New acute medical unit (AMU), which uses the objectives and aims set out by NHS Improvement SDEC. The AEC forms part of new AMU, as a clinic for those patients 'fit to sit', to avoid unnecessary hospital admissions in clinically stable, ambulatory patients.	Implementing an additional senior decision maker improved average time to senior review from 3 hours 19 minutes to 2 hours 56 minutes and subsequently decision to discharge from 3 hours 28 minutes to 2 hours 30 minutes.<br><br>Burden on medical take significantly reduced; 50% of GP expected patients seen by medical take via ED with 9% being reviewed in AEC; after AEC opened 47% seen in AEC. Only 7% reviewed in ED
Weihser et al, 2018	UK (England)<br><br>Single centre<br><br>n=422 (pilot)<br><br>n=501 (mean per month over 1 year)	Observational study	To evaluate enhanced ambulatory care unit model from weekdays only between 08:00 and 18:30) to full-time	Quality improvement intervention: redesign of service incorporating inclusion of POCT; 3-month pilot with limited AEC provision ((operational on weekdays only between 08:00 and 18:30)); full 1-year study with permanent service.<br><br>ED mean LoS, same day discharges, admissions	Redesigned ambulatory emergency care (AEC) pathway including addition of Point of care testing (POCT) for diagnostics. Pathways included the use of National guidance to enable early identification of suitable patients, such as those with chest pain, cellulitis or suspected pulmonary emboli; patient risk stratification through discussion with experienced clinical staff;	Previous AEC service model (a bay within its acute medical unit to provide AEC-style services in conjunction with a specialist nurse-led deep vein thrombosis clinic) saw an average of 25.1 patients per month with ED mean LoS of 250 minutes

					expedited investigations; appropriate treatment and discharge planning controlled and defined by condition-specific patient management algorithms and the involvement of senior decision makers (consultant, registrar or nurse practitioner) at the first point of patient clinical contact	3-month pilot: ED mean LoS for this patient cohort reduced by 40.8% from established baseline of 250 minutes.<br><br>12-month study: ED mean LoS of 115 minutes with an initial clinical review within 13 minutes... a reduction of 54.0% in LoS from the pre-pilot baseline<br><br>**Other ED outcomes:** Conversion rate to admissions, mortality, bed days saved

References:

Dean S, Barratt J. What is the existing evidence base for adult medical Same Day Emergency Care in UK NHS hospitals? A scoping review. *Future Healthcare Journal.* 2024:doi: https://doi.org/10.1016/j.fhj.2024.100011.

Ali A, Karmani J. Audit of a newly developed ambulatory care service at diana princess of wales hospital grimsby UK. *Postgrad Med J* 2018;94:S1.

Atkin C, Knight T, Cooksley T, et al. Performance of admission pathways within acute medicine services: Analysis from the Society for Acute Medicine Benchmarking Audit 2022 and comparison with performance 2019 - 2021. *Eur J Intern Med* 2023;118:89-97.

Edison M, Waskett B, Adekitan D, Behar N. Clinical benefits of a combined physician associate and senior specialist-led emergency surgery ambulatory emergency care clinic introduced in response to the COVID-19 pandemic. *BMJ Open Qual.* 2021;10(4):e001567.

Jarral W, Graf B, Harding S, al. e. Direct referrals from emergency department streaming to ambulatory care: improving same-day emergency care. *Future Healthcare Journal.* 2020;7(S1):s39.

Visanji S, Lyne H, Phillips A, al. e. Using the principles of 'same day emergency care' in our new acute medical unit. *Future Healthcare Journal.* 2020;7(S1):s81.

Weihser P, Giles D. Establishing an ambulatory care service using point-of-care testing diagnostics. *Br J Hosp Med (Lond).* 2018;79(9):520-523.

3. Acute Frailty

Table S3.1: ED waiting time-based metrics (Tier 1)

First author year (ref)	Country & sample size (n=studies)	Study design	Aim	Intervention<br><br>Population	Findings<br><br>Other ED outcomes reported
Hesselink 2019	Total, n=16<br>USA (5), Canada (4), UK (3), Australia (3), Singapore (1)<br>Relevant studies, n=1<br>UK (1)	Systematic review with non-RCT relevant studies<br>Level IV	1) To assess the effectiveness of interventions among older adults in reducing ED crowding<br>2) To identify key characteristics across successful intervention models	'Acute frailty zone' within the ED<br><br>Frail older adults<br>(Frailty not defined)	**ED throughput time:**<br>One controlled before-after study (Taylor, 2016) found that having a dedicated consultant geriatrician in an embedded frailty emergency zone reduced the time to be seen and reviewed as compared with patients receiving emergency care from an 'in-reaching geriatrician service'.<br><br>**Other ED outcomes:** none reported for Taylor 2016 in Hesselink 2019
Huang 2023	Total, n=13<br>USA (4), Ireland (2), Australia (2), UK (1), Spain (1), Sweden (1), Belgium (1), Taiwan (1)<br>Relevant studies, n=6<br>USA (2), UK (1), Belgium (1), Australia (2)	Scoping review with systematic search and non-RCT relevant studies<br>Level IV	To assess models of care for frail older people in the ED	Any model of care 'designed to manage and improve the care of frail older people' within the ED: The Geriatric Emergency Medicine Service model (GEMS); Geriatrician-led Comprehensive Geriatrics Assessment (CGA); MACIAE service; Unplanned Readmission prevention by a Geriatric Emergency Network for Transitional care (URGENT); Geriatric ED Improvement taskforce; Geriatric Emergency Department Intervention model (GEDI)<br><br>Frail older adults (aged ≥65 years)	**ED-LOS:**<br>Four included studies (Argento 2014; Heeren, 2019; Southerland 2018; Wallis, 2018). 'Regarding ED-LOS, three studies reported ED-LOS within the care models ranged from 6 to 15 h (Argento 2014; Heeren, 2019; Southerland 2018). These studies reported reduction in ED-LOS with GEMS, URGENT and the Geriatric ED Improvement taskforce, using two different study designs (case series post-test and sequential cohorts). 'The reduction in ED-LOS ranged from 0.7 h [in Argento 2014; Southerland 2018] to 6.4 h [in Heeren, 2019] for frail older people within the care models' across different study designs. These data appear to relate to average change, excluding the variation.<br>One included study showed a median reduction in ED-LOS with the MACIAE service (n=1130): pre-intervention, 2 days (IQR 3.5) versus intervention, 1.9 days (IQR 3.3) (supplementary material for Shrapnel 2019 in Huang 2023). Outcome was defined as time from ED presentation to admission as an inpatient.<br>Waiting time to be seen by a geriatrician / doctor:<br>According to Huang 2023, one other included study of Geriatrician-led CGA in ED and reported this outcome post-test using a case series; no comparative data were reported. 'Median time seen by a geriatrician from presentation at triage was 1.5 h' (Fox, 2016).

				(Study-level definitions of frailty extracted where reported)	**Other ED outcomes:** death in ED, cost savings per ED presentation, discharge from ED, ED readmission (rate), avoided admission, ward admission, ED revisit
Preston 2018	Total, n=90<br>Relevant studies, n=5<br>UK (3), USA (1)	Systematic mapping review and non-RCT relevant studies<br>Level IV	1. To map interventions that identify frail and high-risk older people in the ED<br>2. To map interventions that manage older people in the ED and the outcomes of these interventions<br>3. To assess evidence of impact of interventions on patient and health service outcomes	Any intervention that identifies and, or manages frail or high-risk older people in the ED: CGA in the ED performed by the OPAL team (older people assessment and liaison); Early CGA in the ED performed by two interface geriatricians; CGA in the ED performed by the GLT (geriatric liaison team); GEDI consultation (geriatric emergency department innovations)/GNL (geriatric nurse liaison intervention) in the ED<br><br>Older people; frail, and or high-risk<br>(Study-level definitions of frailty extracted where reported)	**ED waiting times:**<br>One included study found the '4-hour ED target was achieved in 84.9% [of patients assessed by the OPAL team] and 80.7% of patients [assessed by ED staff alone]' although numbers of patients are not reported (Scott 2014, Wentworth 2015 and Keelan 2016). These data appear to relate to an initial pilot lasting 22 days (Keelan 2016).<br><br>Another included study of Early CGA in the ED reported waiting time as the time waiting to be seen in ED. A 'small reduction in waiting time was seen for patients of all ages' with the intervention as compared with previously (report appendices; Ismail 2014).<br>ED-LOS:<br>'GEDI consultation was associated with a statistically significantly longer median ED-LOS (1.1 hour longer)' in one prospective, observational study (report appendices; Aldeen 2014).<br><br>In another observational study with no apparent control group, 'average LOS in the ED was reduced by 4.8 hours since GLT input became available' for CGA in the ED (report appendices; Thompson 2010).<br>Note that 'length of stay' was not clearly differentiated at times between the hospital and ED.<br><br>**Other ED outcomes:** attendance or reattendance at the ED, ED returns or readmissions, acute admissions from the ED, avoided admissions

Table S3.1.1: ED waiting time-based metrics (Tier 2)

First author year (ref)	Country & sample size (n=studies)	Study design	Aim	Intervention<br><br>Population	Findings<br><br>Other ED outcomes reported
Alakare 2021	Total, n=1<br>Relevant studies, n=1<br>Finland	RCT (randomised controlled trial)<br>Level II	To assess the effects over one year of systematic geriatric assessment + standard care provided in the ED compared to standard care alone on patient and health service outcomes	Systematic geriatric assessment + standard care (n=213) versus standard care alone in the ED (n=219)<br><br>Patients aged 75 years or over who were frail or at risk of frailty.<br><br>Frailty defined by the Clinical Frailty Scale	**ED-LOS:**<br>ED-LOS between groups did not significantly differ: median 7:23 h:minutes (IQR 5:22 − 15:23) and 9:25 h:minutes (IQR 6:00–18:40), for the intervention and the control groups, respectively (P = .05).<br><br>Trend towards average ED-LOS being less with the combined systematic geriatric assessment and standard care compared to standard care alone.<br><br>**Other ED outcomes:** readmission to ED
Kemp 2020	Total, n=1<br>Relevant studies, n= 1<br>Finland	Prospective observational study<br>Level IV	To assess over six months 'the predictive value of the NEWS2 (National Early Warning Score 2) and a three-level ED triage scale' for patient and health service outcomes among frail, older adults	Triage using the NEWS2 grouped as low (0–4), moderate or high (> = 7), and a local three-level triage instrument with levels red (emergent), yellow (urgent) and green (standard) in the ED<br><br>Frail, older adults (aged 75 years or over)<br><br>Frailty defined by a score of at least 4 on the Clinical Frailty Scale (CFS)	**ED length of stay:**<br>Overall mean ED-LOS was 8.6 h (no SD reported); median ED-LOS 6.2 h (no range reported). In 1406 patient visits with a low NEWS2, the mean LOS was 8.67 h (95% CI 8.33–9.02); in 148 patients with a moderate NEWS2, the mean LOS was 8.61 h (95%CI 7.61–9.60) and in 157 patients with a high NEWS2, the mean LOS was 7.48 h (95% CI 6.58–8.39). No statistically significant between-group differences were found (p = 0.095).<br>'Mean LOS for the red group was 4.8 h (95% CI 4.2–5.5), for the yellow group 8.45 h (95% CI 7.8–9.1) and 8.8 h for the green group (95% CI 8.46–9.2)'. A statistically significant difference was found between the red and yellow groups (p < 0.001) but not between the yellow and green groups (p = 0.59). Group size not reported for this outcome.<br><br>**Other ED outcomes:** 72-hour and 30-day ED revisits
Pattinson 2019 (abstract only)	Total, n=1<br>Relevant studies, n=1<br>UK	Pilot service evaluation<br>Level VI	To evaluate over seven weeks the impact of an acute frailty assessment area in the ED	'Six cubicles where frail older patients could be assessed	**ED-LOS:**<br>The 'median length of time to be seen once admitted to frailty area from IAU was 14 minutes.' However, no range in duration or comparative data with standard care reported.

				immediately by the frailty team direct from the initial assessment unit (IAU) in ED using pre-defined frailty criteria'<br><br>Older adults identified as frail in IAU based on predefined (unspecified) frailty criteria	**Other ED outcomes:** 7-day and 28-day ED readmission rates

References:

Alakare J, Kemp K, Strandberg T, et al. Systematic geriatric assessment for older patients with frailty in the emergency department: a randomised controlled trial. BMC Geriatr. 2021;21(1):408.

Hesselink G, Sir Ö, Schoon Y. Effectiveness of interventions to alleviate emergency department crowding by older adults: a systematic review. *BMC Emerg Med.* 2019;19(1):69.

Huang YL, McGonagle M, Shaw R, et al. Models of care for frail older persons who present to the emergency department: A scoping review of the literature. *Int Emerg Nurs.* 2023;66:101250.

Kemp K, Alakare J, Harjola VP, Strandberg T, Tolonen J, Lehtonen L, Castrén M. National Early Warning Score 2 (NEWS2) and 3-level triage scale as risk predictors in frail older adults in the emergency department. BMC Emerg Med. 2020 Oct 28;20(1):83. doi: 10.1186/s12873-020-00379-y

Pattinson J, Kearney F, Azad M, Gladman J, Pitchford R, Ali A. Acute frailty assessment area within the emergency department: a pilot project. Future Healthc J. 2019 Mar;6(Suppl 1):20. doi: 10.7861/futurehosp.6-1-s20

Preston L, Chambers D, Campbell F, Cantrell A, Turner J, Goyder E. *What evidence is there for the identification and management of frail older people in the emergency department? A systematic mapping review.* 2018.

4. In-patient flow interventions (full data too extensive to be reproduced here)

Table S4.1: ED waiting time-based metrics (Tier 1)

First author year (ref)	Country & sample size (n=studies)	Study design	Aim	Intervention<br><br>Population	Outcomes
Anderson, 2022	Total, n=12<br>Australia(3),<br>Belgium (1), Canada (1), Netherlands(1), UK (1),USA (5)<br><br>Relevant studies, n=5, Australia(2),<br>USA(3)	Systematic review and meta-analysis	To evaluate the effectiveness of short-stay, hospital-based mental health crisis units	Short-stay crisis units for mental health patients on crisis care pathways<br>ED patients aged ≥18 years old	ED wait times<br>Time waiting in ED for a bed/transfer<br>Total LOS in ED<br>Total time in crisis and acute care
Beckerleg 2020	Total, n=9<br>Canada(3), S. Korea(3), USA(3)<br>Relevant studies, n=9	Systematic review	To evaluate the impact of workflow interventions on consultation to decision time and ED-LOS in patients referred to consultant services in teaching centres, and to identify barriers to reducing consultation to decision time.	1. SMS messaging reminder for delays in consultation process<br>2. Standardization of the admission process<br>3. Restructuring the consultation process<br>4. Education, audit, and feedback<br>5. Implementation of guidelines<br>6. Modification of staffing patterns<br>Patients ≥ 18 years of age	ED-LOS, Consultation to decision time, and Time from admission decision to patients leaving the ED
Bittencourt 2020	15 Systematic Reviews in total<br>Relevant studies, n=9 (excluded LEAN thinking and physician at triage; and one systematic review from China)	Overview of reviews	To present an overview of systematic reviews on throughput interventions to solve the overcrowding of emergency departments	1. Strengthening of the triage service;<br>2. Strengthening of the ED's team;<br>3. Creation of new care zones;<br>4. Change in ED's work processes<br>General ED population	ED LOS
Burgess 2021	Total, n=26<br>Australia, USA, Netherlands, Hong Kong, Sweden, Canada, Iran, Saudi Arabia (numbers not stated)<br>Relevant studies, n=9 (not stated)	Meta-analysis	To determine the effectiveness of nurse-initiated interventions on patient outcomes in the Emergency Department	Nurse-initiated interventions in ED<br>General ED population	Time-to-treatment in ED
Clark 2022	Total, n=43<br>Countries not stated for all studies	Scoping review	To identify current strategies that hospitals adopt to improve patient bed flow, reduce access	Discharge before noon<br>Discharge lounges<br>Infrastructure (increasing ED beds)	ED Wait times<br>ED-LOS

	Relevant studies, n=6 Australia (1), other(5)		and exit block while optimizing patient care	General ED population	
Considine 2021	Total, n=33<br>Australia(3), Brazil (1), China (3), Denmark(1), Finland(1), Indonesia(2), Italy(2), Netherlands(1), Norway(1), South Korea (1), Singapore (1), Switzerland (1), Taiwan(1), Turkey (8), United Kingdom (4), USA (3)<br>Relevant studies, n=1 (Australia)	Scoping review	To assess the extent, range and nature of published research related to formal systems for recognising and responding to clinical deterioration in emergency department (ED) patients	Use of Rapid Response System to report of clinical deterioration<br>ED patients (⩾18 years) shortness of breath, chest pain or abdominal pain	ED-LOS
Corkery 2021	Total, n=12<br>Australia (3), Canada (1), Jamaica(1). Sweden(1), Switzerland(3), USA(6)<br><br>Relevant studies, n = 11<br>Australia(3), Canada (1), Sweden (1), Switzerland(3), USA(6)<br>(study from Jamaica excluded as non-OECD)	Systematic review	To examine the impact of team triage on waiting times in adult emergency departments	Team triage (a multidisciplinary team sharing responsibilities and duties with a view to improving patient input-throughput measures) vs. nurse- or doctor-led triage<br><br>Patients from convenience sample of adult patients presenting to ED during business hours on Monday and Tuesdays between Sept-Nov 2018, assessed at triage and identified using START tool as likely requiring in-patient admission (START score > 16).<br><br>Fast track only included (MTS category 4–5) patients. Non- English-speaking patients were not included<br><br>Included ambulatory and ambulance patients. Critically ill (MTS category 1) and direct admitted patients (e.g. oncology) were excluded<br><br>Inclusion: adults above 16 years triaged into the emergency room or the waiting room. Exclusion: children<16 years, or patients triaged into urgency care sectors or psychiatry care<br><br>All presenting patients to the ED included. Non-urgent (MTS category 4) patients and acute psychiatric services patients were not included<br><br>All patients presenting during hours when TLP was present were eligible. Excluded pediatric patients (age	ED wait times<br>ED-LOS<br>Time between investigation and treatment<br>Door -to-provider time

				<18 years) frequently seen in nearby dedicated pediatric ED<br><br>All patients presenting into ED, excluding psychiatric complaints or alcohol intoxication<br>No exclusion of patients identified<br>All presenting patients into ED included except children<16 years<br>Exclusion: pregnancy, age younger than 18 years, or any patient that was critically unstable (MTS category 1)	
De Freitas 2018	13 Systematic Reviews in total<br>Relevant studies, n= 10<br>(Villa-Roel, 2012)<br>(excluded senior doctor triage, fast-track, and streaming)	Overview of reviews	[To] provide a comprehensive analysis of the evidence from existing quantitative systematic reviews on the interventions that improve patient flow in EDs	1. Full capacity protocols<br>2. Computerised provider order entry<br>3. Scribes<br>4. Point-of-care testing<br>5. Advanced triage<br>6. Nurse practitioners<br>7. Nurse practitioners/clinical initiative nurse<br>8. Triage nurse ordering<br>9. Nurse-initiated X-rays<br>10. Rapid assessment zones/pods<br>11. Short stay units<br>12. Medical assessment unit<br>General adult ED population	ED LOS<br>Provider to dispatch time<br>Decreased door to physician<br>Physician to disposition decision<br>Disposition decision to discharge times from one study<br>Mean time from medical assessment to decision
Doshmangir 2022	Total, n=64<br>Australia(4), Bahrain(1), Canada (4), China(1), Korea (1), Netherlands(1), Singapore(1), Sweden(3), Tawain(1), UK(3), USA(34)<br>Relevant studies, n=3<br>Canada(2), USA(1)	Systematic review	Investigating initiatives intended to manage adult hospital services and reduce unnecessary hospital use among the general adult population	1. Case management<br>2. Health Information Technologies used with most frequently presenting patients to create individualised care plans for those patients and access to care plans through electronic medical records<br>General adult ED population	ED-LOS
Drennan, 2019	Total, n=16<br>USA(12), Canada(4)<br><br>Relevant studies, n=2, USA(1), Canada(1)	Systematic review	Investigated the impact of Physician Associates on patients' experiences and outcomes, service organisation, working practices, costs and other professional groups for the secondary care specialties of acute medicine, care of the elderly, emergency medicine, trauma and orthopaedics and mental health	Physician Associate (PA) in ED (under the supervision of a physician or as provider of ED care and prescribers of medication)<br>General adult ED population	ED-Wait Times<br>ED-LOS

Dunne 2022	Total, n = 149<br>Australia(5), Canada(10), Japan(2), Kenya(2), Netherlands(3), Spain(2), United Kingdom(2), USA (116), Other (7)<br><br>Relevant studies, n=6 Canada (1), Turkey (1), USA(4)	Systematic review	To summarize the literature regarding the effectiveness of various ED-based interventions to reduce CT usage and identified those that have had the most significant impacts	ED-based intervention to reduce CT utilization<br>Mixed/not specified<br><br>1. Clinical Decision Support and Educational Initiatives<br>2. Patient and Provider Educational Initiatives<br>3. Diagnostic Pathway and Educational Initiatives<br>4. Point of Care Ultrasound (POCUS)<br>5. Provider Performance Feedback<br>6. Specialist Involvement	ED-LOS
Franklin 2020	Total, n=4 (not reported)<br>Relevant studies, n=1 (not reported)	Conceptual review	To provide synthesis of the features, implementation practices, and apparent outcomes (as available) of discharge lounges from which further rigorous investigation may proceed	Discharge lounges<br>General ED population	ED stays >6 hours
Franklin 2022	Total, n=4<br>(USA)<br>Relevant studies, n=1 (USA)	Scoping review	To (i) characterize the general nature of the literature related to CCCs, (ii) synthesize available data regarding their usage and impact, and (iii) discuss the potential mechanisms underlying their impact to facilitate further rigorous investigation	Hospital Capacity Command Centers<br>General ED population	ED wait times<br>ED door to provider time<br>ED left without being seen<br>Patient transport total trip time
Frommer 2022	Total, n = not reported (199 studies assessed for full-text eligibility; Australia, Europe, North America, Japan, Taiwan, Hong Kong, Singapore, South Korea and South America; numbers not reported)<br>Relevant studies, n = 6 Australia(3), USA (1), unclear(2)<br>Type of studies not summarised; Franklin 2020 is a review	Review	To review published literature on solutions to access block	Access block interventions:<br>1. Change the time of discharge<br>2. Discharge lounges<br>3. Short stay units in ED<br>4. Acute medical units (AMUs)<br>5. ED time targets:<br>6. National Emergency Access Target (NEAT)<br>General ED population	In-patient bed waiting times
Gottlieb 2021	Total, n=13<br>(not reported)<br>Relevant studies, n=11 (not reported)	Systematic review	To review the medical literature to determine the utility of triage nurse-ordered testing and to offer evidence-based	Triage nurse-ordered testing<br>General ED population	Wait times<br>ED-LOS

			recommendations to emergency physician		
Grant, 2020	Total, n=99<br>Australia (15), Canada(7), Finland (1), Jamaica(1), Korea(3), Netherlands(3), Pakistan(1), Spain(2), Sweden(2), Switzerland(2), Taiwan(1), Turkey(1), UK(5), USA(55)<br><br>Relevant studies, n=41, Australia(8), Canada(5), Finland(1), Korea(3), Netherlands (1), Switzerland(1), UK(2), USA(20)	Systematic review	To evaluate and summarize the results of studies describing ED throughput interventions	Multiple ED throughput interventions:<br>1. Care of specific patient populations<br>2. Testing strategies<br>3. Alternative ED staffing models<br>4. Other throughput interventions (e.g., CPOE, computerized short messaging service to inform care providers of patient delays, electronic documentation system, physician compensation to reward volume)<br>General ED population/not stated	ED-LOS
Gualandi, 2020	Total, n=38<br>Australia(4), Canada(6), China (1), New Zealand(1), S.Korea(1), Spain (2), Sweden(1), USA (22)<br><br>Relevant studies, n=16<br>Australia(1), Canada (2), S.Korea(1), Spain(1), Sweden(1), USA(10)	Systematic review	To identify actions, actors involved and enablers in improving hospital patient flow	1. Various in-patient flow interventions:<br>2. Introduction of multi-professional teams by reorganization of the work process (each team, consisted of one physician, one Registered Nurse and one Assistant Nurse)<br>3. Implementation of trauma bed protocol in order to faster patient throughput within the ED and decrease trauma admissions to nontrauma ICUs<br>4. Implementation of independent-capacity program with ability of emergency physicians to transfer admitted patients to surrounding area hospitals<br>**5.** Incorporating physician at triage (PAT) and implementation of medical assessment unit (MAU)<br>**6.** Logistic Management Program<br>7. Active bed management in ED, ICU and Coronary Care Unit<br>8. Physician in triage<br>9. Single, centralized Patient Flow Management Center (control of bed management across 3 campuses including service as case management, environmental service, patient transport, ambulance and helicopter dispatch)<br>10. Expansion of ED from 33 to 53 adults bed<br>11. Set of hospital-wide interventions to reduce ED-LOS<br>12. Set of hospital-wide interventions to standardize admission and discharge processes	Wait times<br>Time to physician<br>Total visit time<br>ED-LOS<br>Number of ED patients awaiting hospital bed<br>ED-LOS<br>Boarding time

				13. Set of hospital-wide interventions to reduce ED crowding<br>14. Improvement Team to perform a set of interventions using education, goal setting and real-time performance feedback to improve time to admission to patient referred to general internal medicine<br>15. Set of interventions to improve ED input throughput-output process<br>16. 3 process improvement teams (front-line and<br>17. leadership staff) to focus on discharge, throughput and ED admission<br>18. Collaborative Quality Program to improve ED flow<br>General ED population	
Hesselink 2019	16 included studies, eight (50%) randomized controlled trials (RCTs), two (13%) non-RCTs and six (34%) controlled before-after (CBA) studies.<br>United States (n =5); Canada (n = 4); UK, Australia (n = 3 each); Singapore (n = 1).<br>13 studies (81%) evaluated effects on ED revisits. 4 studies (25%) evaluated effects on ED throughput time. [Mangram et al, 2012, Taylor et al, 2016, Mortimer et al, 2011]	Systematic Review	To assess the effectiveness of interventions on reducing ED crowding by older patients, and to identify core characteristics shared by successful interventions	Interventions for reducing ED crowding by older patients<br>Geriatric pharmacist<br>Older adults (≥ 60 years of age)	ED throughput times<br>EDLOS
Horvath 2023	76 papers; most from United States (n = 28), Australia (n = 25), and Canada (n = 10).<br>Almost three quarters utilized quantitative designs (n=58); qualitative (n= 13) ; mixed methods designs (n= 5).	Scoping Review	To broadly explore and synthesize the current available published and gray literature on the utilization of advanced practice nurses in emergency service models of care, and to learn about development, implementation, patient, provider, and organizational outcomes.	1. Advanced practice nurses<br>2. Generalized emergency models (Advanced practice nurses)<br>3. Minor injury management models (Advanced practice nurses)<br>4. Advanced Nursing practitioner<br>5. Specific population models<br>General ED population (except where stated)	ED wait times<br>ED LOS<br>LWBS<br>Patients discharged within 60 min from initial assessment
Jeyaraman, 2021	139 studies (52%) for ‘outside ED’ interventions or strategies; 129 studies (48%) for ‘within ED’ interventions or strategies	Scoping Review	To identify and summarise literature on the interventions and strategies involving PHCPs (family physicians/general practitioners (GPs), nurse practitioners (NPs) or nurses	Primary healthcare Professionals (NB. “Outside ED” handled in Urgent Community Response section)<br>General ED population	ED LOS<br>LWBS<br>Patient satisfaction<br>Tim to provider initial assessment<br>ED work up time

	41.8% cohort (prospective and retrospective) studies; 8.2% RCTs.<br>North America (n = 156); Europe (n = 76); USA (n = 126).		with expanded role) to manage ED overcrowding.		Leave against medical advice<br>Patient safety<br>Leave before completion of service<br>ED utilisation
Jones 2021	Twenty-one studies met inclusion criteria. 6,7,9,11,18–3412 studies from Australia,6,19,20,25–33 six from New Zealand,7,9,21–24 two from England11,34 and one from Ireland. 18	Systematic review	To provide a synthesis of evidence from studies that address the effect of time-based targets on patient relevant processes and outcomes.	Time-based targets<br>General ED population	Time to assessment<br>Time to in patient team assessments<br>ED LOS<br>Ratio of admitted to discharged patients leaving ED within last 20 min before target was reached
Joseph 2023	9 included papers	Literature review	To assess whether there was a negative or positive outcome following implementation of kiosks in emergency departments.	Kiosks in emergency departments<br>General ED population	ED wait times<br>Time-to-first-identification<br>Usability rate - the percentage of ambulatory patients presenting to ED who used a kiosk during intervention weeks<br>Time-to-triage<br>Time-to-MD
Li 2018	Total, n=137<br>(countries not stated)<br><br>Relevant studies, n=6 (not stated)	Systematic review	To analyze the literature examining the Ambulance Offload Delay (AOD) problem found in journal articles, conference proceedings, grey literature, and books	1. Ambulance offload programs<br>2. Expanding ED capacity<br>General ED population	ED wait times<br>ED LOS<br>Time to see doctor

Lim 2022	Total, n=14<br>Australia(1), Korea(1), UAE(1), USA(11)<br>Relevant studies, n=1<br>Australia(1)	Systematic review	To systematically review the literature for reports of real-time clinical analytics implementation in digital hospitals and to use these findings to synthesize a conceptual framework for LHS implementation	A separate electronic dashboard outside EHR<br>Adult population	ED LOS
Maninchedda 2023	Total, n=19<br>Brazil(1), Belgium(1), Canada(1), China(2), Hong Kong(1) Israel(1), South Africa/UK(1), Taiwan(2), Turkey(1),USA(7), USA/UK(1)<br>Relevant studies, n=1 (country not stated)	Systematic review	To identify the characteristics of the problem, analyzing the proposed strategies aimed at improving patient flow, delay in services provided and overcrowding of emergency departments	Clinical assistance to manage flow<br>General ED population	ED LOS<br>LWBS
Magarey 2023	Total, n=14<br>Australia(9), North America(5)<br>Relevant studies, n=7<br>Australia(4), North America(3)	Systematic review	To investigate the effectiveness of acute short-stay hospital admissions in psychiatric observation units for improving the flow of patients with mental health presentations through<br>the emergency department	Psychiatric observation units<br>Patients presenting to a hospital ED with a psychiatric illness	ED LOS<br>Proportion of patients waiting
Morley 2018	Four prospective studies investigated solutions [Jarvis et al, 2014; Kelen et al, 2001; Shetty et al, 2012; Chang et al, 2018].<br>Four randomised control trials evaluating potential solutions [Holroyd et al, 2007; Jang et al, 2013; Begaz et al, 2017; 25–28; Douma et al, 2016],<br>Remaining studies are mixed-methods or statistical modelling. Most studies from USA (47%), Australia (18%) and Canada (9%)	Systematic review	To critically analyse and summarise the findings of peer-reviewed research studies investigating the causes and consequences of, and solutions to, emergency department crowding.	1. Co-located GP<br>2. The introduction of an ED nurse flow coordinator and increasing bed numbers<br>3. Medical Team Evaluation to improve 'front-end operations' through team triage and a quick registration process<br>4. Early physician assessment<br>5. Use of a flexible-care area<br>6. Adult only population<br>7. Reducing turnaround-time of laboratory tests<br>8. ED nurse flow coordinator<br>9. Expansion of ED<br>10. Bed management<br>11. Leadership programs and leadership support	ED wait times<br>ED LOS<br>ED LOS by patient group<br>LWBS<br>DNW<br>Time taken to turnaround laboratory tests<br>Proportion of patients meeting NEAT time<br>Patients transferred to an inpatient bed within 60 minutes of the decision to admit

				12. Introduction of nationally mandated, timed, patient disposition targets<br>13. Alternative admission units<br>14. Full capacity protocols<br>General ED population	Number of patients leaving the ED within the guideline recommended 4-hours<br>Access block hours
Nguyen 2022	Total of 25 studies<br>3 excluded as paediatric<br><br>22 included studies	Systematic review	To systematically review evidence on the impacts of HISs on patient flow management including what HISs have been used, their application scope, features, and what aspects of patient flow are affected by the HIS adoption.	Health information systems<br>General ED population	Wait times<br>ED LOS<br>Difference between ED exit time and recorded arrival time<br>Arrival to triage<br>Arrival to doctor<br>Doctor to disposition
Pearce 2023	27 Systematic Reviews in total<br><br>Two reviews [16, 30]<br>(Rasouli 2019; Bullard 2012)	Overview of reviews	To analyse clinical research on ED crowding interventions and to summarize which have the best outcomes.	1. Rapid assessment zones<br>2. Nurse ordering imaging and investigations<br>3. Population specific approaches (geriatric)<br>4. Political action<br>5. Full capacity protocol: admitted patients from ED are transported to temporary care spaces<br>6. Medical assessment units<br>7. Providing standards for ED consultant process<br>8. Nurses and paramedics with advanced training utilized in community to triage patients to low acuity settings and provide outpatient support<br>9. Co-localizing primary care within emergency departments<br>10. Patient education<br>11. Use of advanced practice nursing for specific interventions<br>12. Change in physician pay structure to fee for service<br>13. Alternative staffing models<br>General ED population	ED LOS<br>Time to physician assessment<br>LWBS
Sharma 2020	34 articles (31 primary studies and three systematic reviews)<br>(Studies from UK, US, Australia, Canada, and Netherlands)	Systematic review of mixed methods studies	To explore nurses' role(s) and their contribution to maintaining patient flow in acute hospitals through emergency departments.	1. Nurses<br>2. ED ambulance offload nurse<br>3. Mental Health Liaison Nurse (MHLN) service based in emergency department (ED)<br>4. Expanded nursing roles<br>5. Quality Improvement initiative (including ED Flow Coordinator)<br>6. Nurse navigators	ED wait times<br>ED LOS<br>Time to be seen<br>Time seen after triage<br>Time taken for Patient arrival to in-patient<br>NEAT targets

				7. Emergency Journey Co-ordinator<br>8. Nurse navigator role<br>9. ED flow co-ordinator<br>10. Bed Management<br>General ED population	ED time intervals at all stages of the patient journey<br>ED diversion hours<br>Average admission time<br>Transfer time<br>Review time<br>Time of arrival<br>Time to disposition decision<br>Time of transfer to in-patient bed<br>Hold time<br>LWBS
Tampubulon 2018	5 studies in total, two included.	Systematic review	To understand the cause of overflow in order to provide an effective and efficient service to reduce bed overflow and therefore improve patient care in hospitals	Bed Management<br>General ED population	Wait times<br>Time from ED arrival to discharge
Voaklander 2022	35 studies included in review. Majority of included studies conducted in North America (United States n = 12, Canada n = 6) , Smaller number of studies from Asia (South Korea n = 5, Singapore n = 2, Thailand n = 1, Taiwan n = 1, India = 1) and Europe (Ireland n = 1 Turkey n = 2, see Table 1).<br>Majority of studies utilized a before/after study design (n = 25, 71%), of which one study used a controlled before/after design.39<br>Eleven studies (see Table 1 and Appendix S2).17,19,20,29–31,35,36,46,47,51.<br>8/11 assessed impact of interventions to improve	Systematic review	To describe and evaluate effectiveness of interventions aiming to improve ED consultation process.	1. Interventions to improve consult responsiveness<br>2. Interventions to improve access to consultants in the ED<br>3. Interventions to expedite ED consultations<br>4. Interventions to bypass ED consultations and allow emergency physicians to assess and directly admit patients<br>5. Interventions targeting patients with specific conditions<br>General ED population (except where specific condition stated: trauma, pneumonia)	EDLOS<br>Arrival-to-admission<br>Order time<br>Consultation duration<br>Door-to-consultation time<br>Target time met

	consult responsiveness on ED LOS (see Table 2).20,30,31,35,36,46,47,51				

Table S4.2: Ambulance time-based metrics (Tier 1)

First author year (ref)	Country & sample size (n=studies)	Study design	Aim	Intervention<br><br>Population	Findings<br><br>Other ED outcomes reported
Clark 2022	Total, n=43<br>Countries not stated for all studies<br>Relevant studies, n=6<br>Australia(1), other (5)	Scoping review	To identify current strategies that hospitals adopt to improve patient bed flow, reduce access and exit block while optimizing patient care	Infrastructure (increasing ED beds)	Increasing ED beds in a level I trauma center from 28 to 53 did not affect time between ambulance diversions and access to care.(Chrusciel et al, 2019) Single-site study. Data integrity open to question owing to multimodal interventions.<br><br>For other Outcomes see **Tables 18 & 22**.
Franklin 2022	Total, n=4<br>(USA)<br>Relevant studies, n=1<br>(USA)	Scoping review	To (i) characterize the general nature of the literature related to CCCs, (ii) synthesize available data regarding their usage and impact, and (iii) discuss the potential mechanisms underlying their impact to facilitate further rigorous investigation		**Ambulance diversion:** Ambulance diversion (hours per month) decreased by 79 (from86 to 7; 95%CI, 111 decrease, 46 decrease) [23]
Gualandi, 2020	Total, n=38<br>Australia(4), Canada(6),China(1), New Zealand(1), S.Korea(1), Spain(2), Sweden(1), USA(22)<br>Relevant studies, n=16<br>Australia(1), Canada(2), S.Korea(1), Spain(1), Sweden(1), USA(10)	Systematic review	To identify actions, actors involved and enablers in improving hospital patient flow	1. Set of interventions implemented to improve ED input throughput-output process<br>2. Three process improvement teams (front-line and leadership staff) to focus on discharge, throughput and ED admission	1. Decrease in **Ambulance diversion hours**. [Twanmoh 2006 - USA]<br><br>2. **Ambulance diversion hours:** 72% of ambulance diversion hours reduction from 2.365 to 65. [Yancer, 2006 - Canada]
Li 2018	Total, n=137<br>(countries not stated)	Systematic review	To analyze the literature examining the Ambulance Offload Delay (AOD) problem	 <br>Ambulance offload programs	**Ambulance response times: None**<br><br>**Ambulance Offset Delay (AOD):**

	Relevant studies, n=14 (countries not stated)		found in journal articles, conference proceedings, grey literature, and books	Expanding ED capacity<br><br>Unspecified strategies to increase ED throughput<br><br>Ambulance diversion (AD)<br><br>Patient allocation policy<br><br>Redirecting patients to alternative care destinations	Clarey et al. [65] design a discrete event simulation model to assess the change on AOD in a scenario where dedicated nurses were hired to assist with offloading ambulance patients. This study demonstrates a clear reduction in AOD when dedicated nursing levels are increased. However, the authors also raise their concern that using this as a sole method to reduce AOD requires unacceptably low staff utilisation.<br><br>Silvestri et al. (2006) performed 22- month longitudinal observational study between 2003 and 2004, to examine impact of ED bed availability on AOD time in regional EMS system with four hospitals in Orlando, FL, USA and found that an increase in ED bed availability decreases AOD.<br><br>Majedi (2008) models interaction of an EMS and a hospital ED using queuing theory, and tests scenarios such as adding more ED beds, adding more ambulances, and reducing the ED-LOS of patients. Majedi concludes that adding more beds to the ED can have a positive impact on multiple performance measures including average number of ambulances in offload delay, the average AOD, ambulance […] utilization.<br><br>Crilly et al (2014) investigate the impact of opening a new ED on patient and healthcare service outcomes in Queensland, Australia. Healthcare service and patient outcomes at the two preexisting hospitals (including ambulance offload time [..] and access block) did not improve.<br><br>Crilly et al (2020) conducted a retrospective comparative cohort study to identify predictors of admission and to describe outcomes for ambulance patients at three Australian EDs, before and after the opening of 41 additional ED beds (from 81 to 122). The authors report that […] AOD, did not show any improvement.<br><br>Majedi (2008) shows that reducing patients' ED-LOS, which increases ED patient throughput, can have a positive impact on EMS system performance, including the average number of ambulances in AOD.<br><br>Pre−/post- OCP comparison study (McRae et al.(2012)). ED volume increased 7.0% while the ambulance service demand increased 11.1%. Authors report that improvements in ED patient flow led to a reduction in mean AOD.

Li 2018	Total, n=137<br>(countries not stated)<br><br>Relevant studies, n = 1<br>(Baltimore, USA (Halliday et al (2016)))	Systematic review	To analyze the literature examining the Ambulance Offload Delay (AOD) problem found in journal articles, conference proceedings, grey literature, and books	Senior paramedic as re-router	Halliday et al (2016)) assigned senior paramedic to recommend the appropriate transport destinations in the event of ED crowding. They report a 1.35-min decrease of the average at-hospital time from pre- to post-intervention periods, and a 4.53-min decrease from the seasonal match control to post-intervention periods, representing a statistically significant decrease. Study emphasizes importance of better communication and coordination between EMS and hospital EDs as well as future intervention initiatives.
Li 2018	Total, n=137<br>(countries not stated)<br><br>Relevant studies, n = 1<br>(Asmaoah et al, 2008)	Systematic review	To analyze the literature examining the Ambulance Offload Delay (AOD) problem found in journal articles, conference proceedings, grey literature, and books	Limitation Policy for Ambulance Diversion	Asamoah et al. (2008) employ strict limitation policy to reduce AD (restricting each hospital to 1 h out of every 8), and report an 82% reduction in AD. However, they also observe that the mean AOD time increases by 32% as a side effect.<br><br>Schull et al. (2003) demonstrate that AD time increased by 6.2 min per admitted patient boarded in the ED due to ED crowding.
Li 2018	Total, n=137<br>(countries not stated)<br><br>Relevant studies, n = 1<br>(Shah et al, 2006) [U.S.]	Systematic review	To analyze the literature examining the Ambulance Offload Delay (AOD) problem found in journal articles, conference proceedings, grey literature, and books	Voluntary, physician-directed ambulance destination control program	Shah et al. (2006) implement a voluntary, physician-directed ambulance destination control program in Rochester, NY, USA to direct ambulances to the ED that is most able to provide appropriate and timely care. EMS providers are asked to call a destination-control physician for patients requesting transport to either of the two participating hospitals. The physician determines patient destination by using patient and system variables as well as EMS providers/patient input.<br>During the intervention period, 2708 patients were transported to the participating hospitals. EMS providers contacted the destination-control physician for 1866 (69%) patients. The original destination was changed for 253 (14%) patients. AD decreased 190 (41%) hours at the university hospital and 62 (61%) hours at the community hospital, as compared with the control period. The authors conclude that this type of program may be effective in reducing overcrowding and maximizing the availability of emergency health care resources.
Li 2018	Total, n=137<br>(countries not stated)<br><br>Relevant studies, n= 1<br>(Newell et al, 2013; Ottawa, Canada)	Systematic review	To analyze the literature examining the Ambulance Offload Delay (AOD) problem found in journal articles, conference	Offloading without charting	Newell et al. (2013) reports another EMS intervention trialed in Ottawa, ON, Canada where paramedics depart immediately after offloading a patient without charting (CTAS 2,3,4 only). Required electronic paramedic care report then completed by paramedics while mobile and sent to hospital ED via a secure Wi-Fi connection.

			proceedings, grey literature, and books		Average ambulance turnaround time dropped by 14 min per patient transported during eight-week trial period. Despite success, intervention encountered resistance due to patient safety concerns and hospitals having timely access to patient information.
Morley et al 2018	5 studies including leadership programs	Systematic review	To critically analyse and summarise the findings of peer-reviewed research studies investigating the causes and consequences of, and solutions to, emergency department crowding.	Leadership programs and leadership support.	Hospital leaders and ED staff worked collaboratively (U.S.) to introduce a computerised tracking system to ensure the ability for real time tracking of ED admit wait times. The group agreed to measurable goals in terms of the time between the decision to admit and final transfer to an inpatient bed. Study reported significant decrease in hours of ambulance diversion [Patel et al, 2014].
Morley et al 2018<br><br>4 studies	Four prospective studies [Jarvis et al, 2014; Kelen et al, 2001; Shetty et al, 2012; Chang et al, 2018].<br>Four RCTs [Holroyd et al, 2007; Jang et al, 2013; Begaz et al, 2017; 25–28; Douma et al, 2016],<br>Remaining studies mixed-methods or statistical modelling. Most studies from USA, Australia and Canada	Systematic review	To critically analyse and summarise the findings of peer-reviewed research studies investigating the causes and consequences of, and solutions to, emergency department crowding.	Impact of 14-bed monitored inpatient unit, staffed by ED.	Kelen et al (2001) (US) reported significant decreases in hours of ambulance diversion
Morley et al 2018<br><br>4 studies	Four prospective studies [Jarvis et al, 2014; Kelen et al, 2001; Shetty et al, 2012; Chang et al, 2018].<br>Four RCTs [Holroyd et al, 2007; Jang et al, 2013; Begaz et al, 2017; 25–28; Douma et al, 2016],<br>Remaining studies are mixed-methods or statistical modelling. Most studies from USA, Australia and Canada	Systematic review	To critically analyse and summarise the findings of peer-reviewed research studies investigating the causes and consequences of, and solutions to, emergency department crowding.	Full capacity protocols<br><br>Implementation of independent or full capacity program to provide alternative options for admission in times of crowding	Study of relatively new full capacity protocol (U.S.), Reported a 92% significant decrease in hours of ambulance diversion related to the protocol [Willard et al 2017].<br><br>Reported significant 34 minute increase in ED-LOS on days when protocol was operational [Willard et al 2017].
Reay et al 2020	15 studies in total.<br><br>6 intervention studies, 4 with a pre-post design and 2	Systematic review	To (1) explore factors that impact transitions in care from EMS providers to ED nurses,	ED ambulance off load nurse dedicated to triaging and assessing EMS patients.	Patients offloaded if ED treatment space was available, or nurse provided prescribed treatments for Category 3–5 patients (Australian Triage Scale) while patients remained on ambulance stretcher (46). Using administrative data (21,454 patients), this role significantly

	using post-only design; 1 retrospective pre-post study included.		and to (2) identify effective interventional strategies.		improved time for patients to be seen by 2 minutes and reduced number of patients who left ED without being seen. No changes to length of stay. Hospital discontinued role after study had completed.
Sharma et al 2020	34 articles (31 primary studies and three systematic reviews) (Studies from UK, US, Australia, Canada, and Netherlands)	Systematic review of mixed methods studies	To explore nurses' role(s) and their contribution to maintaining patient flow in acute hospitals through emergency departments.	Bed Management - intervention(s) aimed at improving patient flow, time to bed placement, and reducing hold hours.	Barrett et al. , 2012 QI before-after study used ED data for patients (n = 10,967) who received care during 2010. 221-bed Trauma II medical centre USA<br><br>**Reduction in ambulance diversion rates** 11.6% to 0.3%,
Sharma et al 2020	34 articles (31 primary studies and three systematic reviews) (Studies from UK, US, Australia, Canada, and Netherlands)	Systematic review of mixed methods studies	To explore nurses' role(s) and their contribution to maintaining patient flow in acute hospitals through emergency departments.	ED ambulance offload nurse<br><br>ED expanded nursing roles	Greaves et al., 2017 Retrospective case study Data collected for all patient presentations covered equal time periods (39 days) before and after implementation of the EDAOLN role. 570 bed hospital ED in a tertiary Hospital Australia. Data collection from ED Information System for all ED presentations for time to be seen, proportion of hospital admission and number of patients did not wait to be seen. Time to be seen improved marginally during the trial period of the EDAOLN (T1: 34 min vs. T2: 31 min, p = .002). ED-LOS, NEAT and access block did not differ.<br><br>Elder et al., 2015 Systematic review 21 primary studies. 7 studies exploring expanded roles. England (n = 5), Australia (n = 4), Canada (n = 1), Sweden(n = 1) To explore literature regarding three key strategies designed to promote patient throughput in the ED<br><br>Outcomes for ambulance diversion and patient off stretcher times (POST).

References:

Anderson, K., Goldsmith, L.P., Lomani, J., Ali, Z., Clarke, G., Crowe, C., Jarman, H., Johnson, S., McDaid, D., Pariza, P. and Park, A.L., 2022. Short-stay crisis units for mental health patients on crisis care pathways: systematic review and meta-analysis. BJPsych open, 8(4), p.e144.

Beckerleg, W., Wooller, K. and Hasimjia, D., 2020. Interventions to reduce emergency department consultation time: A systematic review of the literature. Canadian Journal of Emergency Medicine, 22(1), pp.56-64.

Bittencourt, R. J., Stevanato, A. D. M., Bragança, C. T. N., Gottems, L. B. D., & O'Dwyer, G. (2020). Interventions in overcrowding of emergency departments: an overview of systematic reviews. Revista de saúde publica, 54, 66.

Burgess, L. and Kynoch, K., 2021. Effectiveness of nurse-initiated interventions on patient outcomes in the emergency department: a systematic review protocol. JBI Evidence Synthesis, 15(4), pp.873-881.

Clark, J., & Islam, M. S. (2022). Hospital Access Block: A Scoping Review. Journal of Emergency Nursing, 48(4), 430-454.

Considine, J., Fry, M., Curtis, K., & Shaban, R. Z. (2021). Systems for recognition and response to deteriorating emergency department patients: a scoping review. Scandinavian journal of trauma, resuscitation and emergency medicine, 29(1), 69. https://doi.org /10.1186/s13049-021-00882-6

Corkery, N., Avsar, P., Moore, Z., O'Connor, T., Nugent, L., & Patton, D. (2021). What is the impact of team triage as an intervention on waiting times in an adult emergency department?–A systematic review. International Emergency Nursing, 58, 101043.

De Freitas, L., Goodacre, S., O'Hara, R., Thokala, P., & Hariharan, S. (2018). Interventions to improve patient flow in emergency departments: an umbrella review. Emergency Medicine Journal, 35(10), 626-637.

Doshmangir, Leila, Roghayeh Khabiri, Hossein Jabbari, Morteza Arab-Zozani, Edris Kakemam, and Vladimir Sergeevich Gordeev. "Strategies for utilisation management of hospital services: a systematic review of interventions." Globalization and Health 18, no. 1 (2022): 53.

Drennan, Vari M., Mary Halter, Carly Wheeler, Laura Nice, Sally Brearley, James Ennis, Jon Gabe et al. "The role of physician associates in secondary care: the PA-SCER mixed-methods study." Health services and delivery research 7, no. 19 (2019): 1-158.

Dunne, C. L., Elzinga, J. L., Vorobeichik, A., Sudershan, S., Keto-Lambert, D., Lang, E., & Dowling, S. (2022). A systematic review of interventions to reduce computed tomography usage in the emergency department. Annals of Emergency Medicine, 80(6), 548-560.

Franklin, B. J., Mueller, S. K., Bates, D. W., Gandhi, T. K., Morris, C. A., & Goralnick, E. (2022). Use of hospital capacity command centers to improve patient flow and safety: a scoping review. Journal of Patient Safety, 18(6), e912-e921.

Franklin, B. J., Vakili, S., Huckman, R. S., Hosein, S., Falk, N., Cheng, K., ... & Goralnick, E. (2020). The inpatient discharge lounge as a potential mechanism to mitigate emergency department boarding and crowding. Annals of Emergency Medicine, 75(6), 704-714.

Frommer, M., & Marjanovic, S. (2022). Access block: a review of potential solutions. Glebe (AU): Sax Institute.

Gottlieb, M., Farcy, D. A., Moreno, L. A., Vilke, G. M., & Guittard, J. A. (2021). Triage nurse-ordered testing in the emergency department setting: a review of the literature for the clinician. The Journal of Emergency Medicine, 60(4), 570-575.

Grant, Kiran L., Conrad J. Bayley, Zahra Premji, Eddy Lang, and Grant Innes. "Throughput interventions to reduce emergency department crowding: a systematic review." Canadian Journal of Emergency Medicine 22, no. 6 (2020): 864-874.

Gualandi, Raffaella, Cristina Masella, and Daniela Tartaglini. "Improving hospital patient flow: a systematic review." Business process management journal 26, no. 6 (2020): 1541-1575.

Hesselink, G., Sir, Ö., & Schoon, Y. (2019). Effectiveness of interventions to alleviate emergency department crowding by older adults: a systematic review. BMC emergency medicine, 19, 1-9.

Horvath, S., Visekruna, S., Kilpatrick, K., McCallum, M., & Carter, N. (2023). Models of care with advanced practice nurses in the emergency department: A scoping review. International Journal of Nursing Studies, 104608.

Jeyaraman, M. M., Copstein, L., Al-Yousif, N., Alder, R. N., Kirkland, S. W., Al-Yousif, Y., ... & Abou-Setta, A. M. (2021). Interventions and strategies involving primary healthcare professionals to manage emergency department overcrowding: a scoping review. BMJ open, 11(5), e048613.

Jones, P., Haustead, D., Walker, K., Honan, B., Gangathimmaiah, V., Mitchell, R., ... & Mountain, D. (2021). Has the implementation of time-based targets for emergency department length of stay influenced the quality of care for patients? A systematic review of quantitative literature. Emergency Medicine Australasia, 33(3), 398-408.

Joseph, M. J., Summerscales, M., Yogesan, S., Bell, A., Genevieve, M., & Kanagasingam, Y. (2023). The use of kiosks to improve triage efficiency in the emergency department. NPJ Digital Medicine, 6(1), 19.

Li, M., Vanberkel, P., & Carter, A. J. (2019). A review on ambulance offload delay literature. Health care management science, 22(4), 658-675.

Lim, H. C., Austin, J. A., Van Der Vegt, A. H., Rahimi, A. K., Canfell, O. J., Mifsud, J., ... & Sullivan, C. M. (2022). Toward a learning health care system: a systematic review and evidence-based conceptual framework for implementation of clinical analytics in a digital hospital. Applied Clinical Informatics, 13(02), 339-354. DOI: 10.1055/s-0042-1743243

Magarey, A. W., Weng, J., Looi, J. C., Allison, S., & Bastiampillai, T. (2023). Systematic Review of Psychiatric Observation Units and Their Impact on Emergency Department Boarding. The Primary Care Companion for CNS Disorders, 25(6), 49818.

Maninchedda, Mario, Anna Silvia Proia, Lavinia Bianco, Mariarosaria Aromatario, Giovanni Battista Orsi, and Christian Napoli. "Main Features and Control Strategies to Reduce Overcrowding in Emergency Departments: A Systematic Review of the Literature." Risk Management and Healthcare Policy (2023): 255-266.

Morley, C., Unwin, M., Peterson, G. M., Stankovich, J., & Kinsman, L. (2018). Emergency department crowding: a systematic review of causes, consequences and solutions. PloS one, 13(8), e0203316.

Nguyen, Quy, Michael Wybrow, Frada Burstein, David Taylor, and Joanne Enticott. "Understanding the impacts of health information systems on patient flow management: A systematic review across several decades of research." PloS one 17, no. 9 (2022): e0274493.

Pearce, S., Marr, E., Shannon, T., Marchand, T., & Lang, E. (2023). Overcrowding in emergency departments: an overview of reviews describing global solutions and their outcomes. Internal and Emergency Medicine, 1-9.

Reay, G., Norris, J. M., Nowell, L., Hayden, K. A., Yokom, K., Lang, E. S., ... & Abraham, J. (2020). Transition in care from EMS providers to emergency department nurses: a systematic review. Prehospital emergency care, 24(3), 421-433.

Sharma, S., Rafferty, A. M., & Boiko, O. (2020). The role and contribution of nurses to patient flow management in acute hospitals: A systematic review of mixed methods studies. International Journal of Nursing Studies, 110, 103709.

Tampubolon, L. (2018). Bed Management Strategy for Overcrowding at the Emergency Department: A Systematic Review. KnE Life Sciences, 50-59.

Voaklander, B., Gaudet, L. A., Kirkland, S. W., Keto-Lambert, D., Villa-Roel, C., & Rowe, B. H. (2022). Interventions to improve consultations in the emergency department: A systematic review. Academic Emergency Medicine, 29(12), 1475-1495.

5. Care Transfer Hubs

Table S5.1: ED waiting time-based metrics (Tier 1)

First author year (ref)	Country & sample size (n=studies)	Study design	Aim	Intervention<br><br>Population	Findings<br><br>Other ED outcomes reported
Birtwell, 2022	N=19 (total)<br><br>Relevant: 2 (Australia: 2)	Systematic review of meta-analysis	To assess the effects of TCIs on patient-related outcomes transitioning into and out of LTCFs.	Interventions: TCIs (e.g., early Assessment post-discharge interventions, acute nursing support, pharmacist transition coordinated interventions, outreach & nurse-led telephone triage lines, education & care plans' implementation, multi-disciplinary teams in care management)<br><br>Population: LTCFs residents in transition from hospitals to LTFCs or from LTFCs to hospitals.	**ED-LOS**<br>ED-LOS significantly decreased for intervention groups (3 studies; standardized mean difference, −3.00 [95%CI, −3.61 to −2.39]; I2 = 99% [95%CI, 98%-99%]).<br><br>**Other ED outcomes:** Hospital readmission, ED readmission, functional independence, QoL
van den Broek, 2023	N=12 (total)<br><br>Relevant: 3 (Belgium: 1; UK: 1; USA: 1)	Systematic review	To assess the impact of ED-based interventions	Interventions: ED-based interventions delivered by healthcare professionals specialised to transitional care (i.e., ED-nurse discharge service, nurse-led transitional care, care coordination team, patient navigator).<br><br>Population: older adults (aged over 65)	**ED-LOS**<br>One study reported no statistically significant effects of needs assessments performed by ED discharge nurses on ED-LOS. ED-LOS, however, decreased significantly more than 6 hours after the needs assessment by a geriatric ED nurse.<br><br>**Other ED outcomes:** ED revisit, hospital readmission, patient transition outcomes

References:

Birtwell K, Planner C, Hodkinson A, et al. Transitional Care Interventions for Older Residents of Long-term Care Facilities: A Systematic Review and Meta-analysis. *JAMA Netw Open.* 2022;5(5):e2210192.

van den Broek S, Westert GP, Hesselink G, Schoon Y. Effect of ED-based transitional care interventions by healthcare professionals providing transitional care in the emergency department on clinical, process and service use outcomes: a systematic review. *BMJ Open.* 2023;13(3):e066030.

6. Community beds

No evidence

References:

None

7. Intermediate care

Table S7.1: ED waiting time-based metrics (Tier 1)

First author year (ref)	Country & sample size (n=studies)	Study design	Aim	Intervention<br><br>Population	Findings<br><br>Other ED outcomes reported
Birtwell, 2022	N=19 (total)<br><br>Relevant: 14 (Australia, Denmark, USA)	SR (with MA)	To assess the effects of TCIs on patient-related outcomes transitioning into and out of LTCFs.	Interventions: TCIs (e.g., early Assessment post-discharge interventions, acute nursing support, pharmacist transition coordinated interventions, outreach & nurse-led telephone triage lines, education & care plan implementation, multi-disciplinary teams in care management)<br><br>Population: LTCFs residents in transition from hospitals to LTFCs or from LTFCs to hospitals.	Meta-analysis results (SMD, 95%CI)<br>- TCIs (studies=3; outcome: LOS in ED): **−3.00 (−3.61, −2.39)**, I^2 = 99%<br>- TCIs (studies=7; outcome: LOS in hospital): −1.86 (−5.47, 1.75), I^2 = 98%<br>- TCIs (studies=2; outcome: QoL): −0.04 (−0.46 to 0.38), I^2 = 92%
Totten, 2019	N=233 (total)<br><br>Relevant: (Australia/New Zealand: 19, Canada: 4, UK: 22, USA: 110)	SR (with MA)	To assess the effects of telehealth consultations on intermediate care outcomes, costs, QoL.	Interventions: Inpatient telehealth consultations<br><br>Population: Patients (no restrictions applied)	- Tele-stroke services not effective in 3-month mortality (SoE: Moderate); Specialty consultations in ED showed generally positive effects on mortality (not statistically significant results) (SoE: Low), **generally positive effects on waiting times in ED** (SoE: Moderate) and savings (SoE: Low).
van den Broek, 2023	N=12 (total)<br><br>Relevant: 12 (Australia, Belgium, Canada, UK, USA)	SR	To assess the impact of ED-based interventions	Interventions: ED-based interventions delivered by healthcare professionals specialised to transitional care (i.e., ED-nurse discharge service, nurse-led transitional care, care coordination team, patient navigator).<br><br>Population: older adults (aged over 65)	LOS (ED/hospital) (3 studies): One study found a statistically significant effect of ED-based interventions in reducing LOS, one study found statistically significant effect of control/pre-intervention group in reducing LOS, and one study reported NS results.

References:

Birtwell K, Planner C, Hodkinson A, et al. Transitional Care Interventions for Older Residents of Long-term Care Facilities: A Systematic Review and Meta-analysis. JAMA Netw Open. 2022;5(5):e2210192.

Totten AM, Hansen RN, Wagner J, et al. AHRQ Comparative Effectiveness Reviews. In: Telehealth for Acute and Chronic Care Consultations. Rockville (MD): Agency for Healthcare Research and Quality (US); 2019.

van den Broek S, Westert GP, Hesselink G, Schoon Y. Effect of ED-based transitional care interventions by healthcare professionals providing transitional care in the emergency department on clinical, process and service use outcomes: a systematic review. BMJ Open. 2023;13(3):e066030.

8. Single Point of Access (SPoA)

Table S8.1: ED waiting time-based metrics (Tier 1)

First author year (ref)	Country & sample size (n=studies)	Study design	Aim	Intervention<br><br>Population	Findings<br><br>Other ED outcomes reported
Anderson et al (2022)	Twelve studies across six countries (Australia, Belgium, Canada, The Netherlands, UK, USA)<br><br>67,505 participants	Systematic Review and Meta-Analysis	To evaluate the effectiveness of short-stay, hospital-based mental health crisis units.	Short-stay crisis units for mental health patients on crisis care pathways	ED-LOS<br>Meta-analysis indicated significant reduction in length of emergency department stay (by 164.24 min; 95% CI -261.24 to -67.23 min; P < 0.001) and number of in-patient admissions (odds ratio 0.55, 95% CI 0.43-0.68; P < 0.001).<br><br>Other Outcomes:<br>Units could reduce psychiatric holds (42% after vs. 49.8% before intervention; difference = 7.8%; P < 0.0001) and increase out-patient follow-up care ($\chi 2$ = 37.42, d.f. = 1; P < 0.001).<br><br>Concludes that short-stay mental health crisis units are effective for reducing emergency department wait times and in-patient admissions. Further research should investigate the impact of units on patient experience, and clinical and social outcomes.
Gonçalves-Bradley et al (2018)	Studies from Ireland, the UK, and Australia	Cochrane Review	To assess effects of locating primary care professionals in hospital EDs to provide care for patients with non-urgent health problems, compared with care provided by regularly scheduled emergency physicians (EPs).	Introducing GPs or emergency NPs to provide care to patients with non-urgent problems in the ED, as compared to EPs for outcomes such as resource use.	Identified four trials (one RCT and three non-randomised trials), involving 11,463 patients, 16 general practitioners (GPs), 9 emergency nurse practitioners (NPs), and 69 Eps. Studies had an overall high or unclear risk of bias. Outcomes investigated similar across studies. Considerable variation in triage system used, level of expertise and experience of the medical practitioners, and type of hospital. High heterogeneity across studies precluded pooling data.<br>Uncertain whether intervention reduces time from arrival to clinical assessment and treatment or total length of ED stay (1 study; 260 participants) with evidence of very low-certainty.<br>Assessed evidence from four included studies of very low-certainty. Results are inconsistent. Safety has not been examined. Evidence is insufficient for conclusions for practice or policy regarding effectiveness and safety of care provided to non-urgent patients by GPs and NPs versus EPs in the ED to mitigate problems of overcrowding, wait times, and patient flow.
Jeyaraman, 2021	139 studies (52%) for 'outside ED' interventions or strategies;	Scoping Review	To identify and summarise literature on the interventions and strategies	Primary healthcare Professionals (NB. "Outside ED" handled in Urgent Community Response section)	Studies reporting 'within ED' interventions or strategies investigate impact on 'through-put'-related outcomes (i.e., ED patient flow metrics). Majority of included studies report positive impact on ED-LOS (73.6%). Remainder reported either negative/no impact.

	129 studies (48%) for ‘within ED’ interventions or strategies<br>41.8% cohort (prospective and retrospective) studies; 8.2% RCTs.<br>North America (n = 156); Europe (n = 76); USA (n = 126).		involving PHCPs (family physicians/general practitioners (GPs), nurse practitioners (NPs) or nurses with expanded role) to manage ED overcrowding.	General ED population	Majority of studies reported ED outcomes (e.g. ED visits, time to provider initial assessment, number of patients diverted to primary care and ED cost savings. About 62% of included studies reported a positive impact on ED utilisation (ie, decrease in lower acuity ED visits). 28% reported no impact and 9.7% reported negative impact (increased ED utilisation).
Jeyaraman, 2022	40 comparative studies including 10 RCTs and 13 pre–post studies.<br>30 studies (75%) reported ED-LOS	Systematic review and meta-analysis	To identify, critically appraise and summarise evidence on impact of employing primary healthcare professionals (PHCPs: family physicians/general practitioners (GPs), nurse practitioners (NP) and nurses with increased authority) in emergency department (ED) triage, on patient flow outcomes.	Primary Healthcare Professionals<br>General ED population	Length of stay (ED-LOS) was included in secondary outcomes. Eight RCTs (six of moderate quality and two of low quality), reported significant decrease in ED-LOS (MD −15.31 min (95% CI −18.35 to −12.27); eight studies; I2: 0%; p<0.00001) in PHCP triage intervention (nurse triage-plus) group vs. traditional nurse-led triage model.<br>The CBA studies (low quality) reported a significant decrease in ED-LOS (mean difference −63.17 min (95% CI −101.93 to −24.40); three studies; I2: 51%; p=0.001) in PHCP triage intervention group (two nurse-triage plus and one NP team triage) vs. traditional nurse-led triage model.<br>Three retrospective cohorts (low quality) also reported significant decrease in ED-LOS (MD −13.96 min (95% CI −19.31 to −8.61); three studies; I2: 37%; p<0.00001) in the PHCP triage intervention group (nurse triage-plus) vs. traditional nurse-led triage model.
Magarey 2023	Total, n=14<br>Australia(9), North America (5)<br>Relevant studies, n=7<br>Australia(4), North America(3)	Systematic review	To investigate effectiveness of acute short-stay hospital admissions in psychiatric observation units for improving the flow of patients with mental health presentations through	Psychiatric observation units	ED-LOS:<br>Three North American studies demonstrated changes in ED-LOS for psychiatric patients.([Kim 2022; EmPATH; Pre- and post- intervention retrospective; Stamy 2021; EmPATH; Pre- and post- intervention retrospective, Parwani 2018; Crisis intervention unit; Pre- and post-intervention retrospective). All 3 studies showed positive impact on reducing ED-LOS, the biggest reduction being 11 hours and 18 minutes, (Kim 2022) followed by a reduction of roughly 2 hours for the other 2 studies.[Stamy 2021, Parwani 2018] Kim et al (2022) found ED-LOS became more predictable and less varied after implementing EmPATH unit. Stamy et al (2021) reported fewer incomplete admissions after EmPATH implementation.

			the emergency department	Patients presenting to a hospital ED with a psychiatric illness	ED-LOS:<br>2/3 of Australian studies that stated ED-LOS showed significant improvement in ED-LOS, (Braitberg 2018; pre- and post-intervention retrospective case control; Huber 2021; Psychiatric emergency care centre; retrospective case series). Mitchell et al (2020) (mixed prospective and retrospective case control; psychiatric assessment and planning over 3 sites), showed improved ED-LOS at only 1 site.<br><br>Proportion of patients waiting:<br>One study [Browne 2011; retrospective case control; psychiatric assessment and planning unit] stated that introduction of the psychiatric observation unit reduced proportion of patients waiting in ED >24 hours and improved 8-hour admission rate.<br>Five studies [Kim 2022; Stamy 2021, Braitberg 2018, Huber 2021, Browne 2011] had serious overall risk of bias (due to confounding); two studies [Parwani 2018]. Mitchell 2020 with moderate overall risk of bias.
Romeo, 2020	86 publications. 19 literature and systematic reviews<br>(Australia 10; Belgium 3; Canada 5;Chile 1; Finland 1; France 1; Global 3; Italy 2; Netherlands 12; Saudi Arabia 1; Spain 1; Sweden 2; Switzerland 4; UK 33; USA 7)	Rapid review	To inform ED strategic decision-making and service design.	Utilisation of General Practitioner (GP) within an ED or closely located unit	Khangura et al. (2012) (S/R) examined effects of locating primary care professionals in the hospital. They concluded with “insufficient evidence upon which to draw conclusions for practice or policy regarding the effectiveness and safety of care provided to non-urgent patients by GPs versus EPs in the ED to mitigate problems of overcrowding, wait-times and patient flow.” Review has been superseded by Gonçalves-Bradley et al (2018) summarised above.<br>Bosmans et al. (2012) (B/A) found that involving a GP on the ED staff and triage by a nurse was more effective than usual care in terms of waiting time, treatment time, without decrease in quality of diagnosis.<br>Van Gils-van Rooij (2018) (O/S) observed that UCCs do not generally enhance the efficiency of patient flow. Median length of stay and wait-times were significant longer in UCCs.<br><br>van Veelen et al (2016) (B/A) found that introduction of GP cooperative with ED resulted in 20% reduction of ED patients. No improved throughput of the remaining patients in the ED (LOS of remaining patients at the ED increased). ED caseload changed, with more referred, complex patients with fewer patient visits in total.

Table S8.1.1: ED waiting time-based metrics (Tier 2)

First author year (ref)	Country & sample size (n=studies)	Study design	Aim	Intervention<br><br>Population	Findings<br><br>Other ED outcomes reported
Bessert et al (2023)	University Medical Center Hamburg-Eppendorf (UKE), Germany.	Prospective, single-center pre-post comparative study. ED patient collective consisted of adult walk-in patients who presented to ED (4 pm – midnight). "Pre" period from August - September 2019, "Post" period from November 2019 (after opening of the WIC) to January 2020.	To study change in utilization of a hospital emergency department (ED) by low-urgency patients after an urgent care walk-in clinic (WIC) was opened in immediate vicinity.	Urgent care walk-in clinic (WIC) (ED patient collective)	4765 ED walk-in patients and 1201 WIC patients. 956 (80.5%) of WIC patients had been referred onward to WIC after initially presenting to the ED; from this group, 790 patients (82.6%) received definitive care in WIC. For patients presenting without any referral document, mean length of stay fell by mean of 17.6 [7.4; 27.8] minutes from its "pre" value of 172.3 minutes.<br>GP-led urgent care walk-in clinic next door to an interdisciplinary hospital emergency department is resource-saving option for walk-in patients who present to ED. Most patients referred from ED to WIC were able to receive definitive care there.
Henderson et al 2020	Australia	Prospective observational study	To identify: (1) whether an after-hours emergency department (ED) collaborative care service using primary contact physiotherapists (PCPs) improves treatment times for musculoskeletal and simple orthopaedic presentations; and (2) differences in	After-hours emergency department (ED) collaborative care service using primary contact physiotherapists (PCPs)<br><br>Diagnosed, matched patients seen in 4-day week after-hours ED primary contact physiotherapy service in tertiary referral ED. Patients presenting with musculoskeletal or simple orthopaedic diagnosis	Patients reviewed by physiotherapist as either primary or secondary physiotherapy contact (1630 - 2030 hours Saturday to Tuesday) included in analysis. Mean (+/-s.d.) treatment time for patients seen by ED PCP was 130+/-76min, compared with 240+/-115min for those seen by a secondary contact physiotherapist (P<0.001).<br><br>Study demonstrates reductions in ED treatment times in after-hours setting for patients managed by an ED PCP. This was achieved by physiotherapists with less reported experience.

			orthopaedic referral rates and analgesia prescription for patients managed by PCPs compared with secondary contact physiotherapists.		
Scantlebury et al (2022)	England.<br>Data from 64 EDs including: non-participant observation of 142 clinical encounters; 467 semistructured interviews with policy-makers, service leaders, clinical staff, patients and carers. Retrospective observational analysis of routinely collected Hospital Episode Statistics. Information on GPED service hours (40 hospitals).	Mixed-methods study: interviews with service leaders and NHS managers; in-depth case studies (n=10) and retrospective observational analysis of routinely collected national data.	To examine effect of general practitioners (GPs) working in or alongside emergency department (GPED) on patient outcomes and experience, and the associated impacts of implementation on the workforce.	Improved access to GP services for people attending EDs either by co-locating GP services alongside EDs at hospital sites, or by employing GPs to work within EDs to see selected patients.	No statistically significant improvement in key performance indicators across several domains including the '4-hour target', hospital admissions and patient outcomes and experience (patients leaving ED without being seen and mortality at 30 days after an ED attendance).<br>Assuming only one GP is present and including salary costs of the GP alone (potentially a substantial underestimate), this amounts to around £454 000 per ED per year. Concludes that current GPED models do not appear to be an efficient use of healthcare resources (Benger et al, 2022).<br>Also included literature review see below.
Wackers et al 2023	Netherlands<br>610 845 patients.	Quasi-experimental study using claims data	To assess whether acute care collaborations (ACCs) are associated with decreased ED utilization, hospital admission rates, and lower costs per patient journey, compared with stand-alone facilities.	Acute care collaborations (ACCs)<br>Patients in Netherlands (2017).	Patient visits in ACCs vs. stand-alone EDs and AHPCs. Number of comorbidities similar in both groups. Multiple logistic and gamma regressions used to determine whether patient visits to ACCs were negatively associated with ED utilization, hospital admission rates, and costs. Logistic regression analysis did not find association between patients visiting ACCs and ED utilization compared to patients visiting stand-alone facilities [odds ratio (OR), 1.01; 95% confidence interval (CI), 1.00-1.03]. However, patients in ACCs associated with increased hospital admissions (OR, 1.07; 95% CI, 1.04-1.09). ACCs associated with higher total costs incurred during patient journey (OR, 1.02; 95% CI, 1.01-1.03). Collaboration between EDs and AHPCs not associated with ED utilization, but associated with increased hospital admission rates, and higher costs. ACCs do not seem to improve health system financial sustainability.

Van der Baaren et al (2022)	Netherlands<br>2772 ED patients presenting with minor traumatic injury	Retrospective cohort study (1 year before; 1 year after)	To evaluate association of ECAP implementation with incidence, severity and length of stay of minor traumatic injuries in ED	Emergency Care Access Point (ECAP)<br><br>General Practice Cooperatives (GPCs)	ED-Length of Stay<br>Median length of stay (hh:mm, IQR) [Injuries] 01:23 (00:58–01:50) 01:19 (00:57–01:49) (p = 0.41)<br>Median length of stay (hh:mm, IQR) [Wounds] 01:00 (00:38–01:31) 01:11 (00:48–01:31) (p = 0.08)<br>Currently, patients referred to ED are assessed twice: in GPC first and then in ED. From patient perspective, this means longer length of stay. On a macro-economic scale, this possibly results in higher total healthcare-related costs because both services are used.

Table S8.2: Ambulance time-based metrics (Tier 1)

First author year (ref)	Country & sample size (n=studies)	Study design	Aim	Intervention<br><br>Population	Findings<br><br>Other ED outcomes reported
Burrell et al, 2023	21 studies (Norway 11; UK 4; Sweden 3; Belgium 1; Switzerland 1; New Zealand 1)	Systematic mapping review and narrative synthesis	To collate and summarise evidence on how GPs are utilised in EMS.	Utilisation of GPs in non-critical EMS<br>GP dispatcher (1) GP attends directly from dispatch (3) GP alerted by dispatcher — GP discretion whether to attend/provide advice (9)<br>Referral to GP by ambulance staff for management at home under supervision of primary care (1) Referral to GP by ambulance staff for transport to primary healthcare centre (2) Referral to GP by ambulance staff for face-to-face consultation (3) Referral to GP by ambulance staff for telephone support (2)	Non-conveyance to ED and hospital admission avoidance were most reported outcome measures for GPs in EMS interventions. The AVS successfully diverted 60.9–77.6% of patients from ED including up to 30 days after AVS contact (Blodgett et al, 2017; 2020).<br>Ambulance duty cycle time for a GP AVS referral was, on average, 15 minutes less than for missions resulting in direct ED conveyance (Blodgett et al, 2017). In Sweden, patients referred to a GP by the EMS triaging nurse were significantly less likely to be transferred to ED (17.4% versus 53.1%, P<0.001) and admitted to hospital (11.4% versus 25.6%, P<0.001), with a significantly shorter mean ambulance mission time (86.88 versus 94.12 minutes, P = 0.04) (Larsson et al, 2017).<br>For the WMAS GP supported assessment scheme, only 21% of patients conveyed to ED compared with 61% for ambulance service as a whole. Patients who received telephone input rather than a face-to-face assessment from a GP were more likely to be conveyed (odds ratio [OR] 2.14, 95% confidence interval [CI] = 1.69 to 2.72) (Villareal et al, 2017). When GPs on-call were not alerted, patients were twice as likely to be transported directly to hospital (31% versus 16%) (Zakariassen & Hunskaar,2010).<br>No included studies reported longer-term clinical outcomes or validated patient-reported experience or outcome measures. None reported a health economic analysis.

Table S8.2.1: Ambulance time-based metrics (Tier 2)

First author year (ref)	Country & sample size (n=studies)	Study design	Aim	Intervention<br><br>Population	Findings<br><br>Other ED outcomes reported
Noble et al (2023)	UK	Conference Abstract for Pilot Study	NR	Working with trust clinical communication centre (CCC) as single point of access, ambulances called before conveying patients within pathways.<br><br>Worked with consultants from Cardiology, Respiratory, Frailty, Emergency Medicine and Acute Medicine to offer senior decision maker input to pre-hospital conversations to define best urgent care pathways.<br><br>Engaged with community falls car, urgent care response team and GPs along with hospital SDECs /virtual wards and provided specialty 'hot clinic' appointments as appropriate, to provide alternatives to ED and admission.<br><br>Used daily huddles to enact rapid cycle PDSA changes.	Pathways with largest impact potential - chest pain, dyspnoea and falls/frailty/head injury. Pathways could also be utilised by GPs referring via CCC, and ED where a patient could be given an alternative to admission.<br><br>Across the pilots, 32-38% given an alternative to ED attendance or admission. 24% avoided hospital entirely.<br><br>**Reduced ambulance lost minutes by 84-87 hours compared to previous 3 week average.**<br><br>Streamlining access to urgent care pathways with a single point of access benefits these patients but also those who do attend by reducing harm from overcrowding through better ED and hospital flow by offering alternatives to ED attendance and admission.

References:

Anderson K, Goldsmith LP, Lomani J, et al. Short-stay crisis units for mental health patients on crisis care pathways: systematic review and meta-analysis. BJPsych Open. 2022;8(4):e144.

Baaren Van Der, R., Barten, D. G., Osch, F. V., Barneveld, K. W. Y. V., Janzing, H., & Cals, J. (2022). Minor Traumatic Injuries In The Emergency Department Pre‐And Post‐Implementation Of An Emergency Care Access Point. Journal Of Evaluation In Clinical Practice, 29(1), 32-38.

Bessert B, Oltrogge-Abiry Jh, Peters Ps, Et Al. Synergism Of An Urgent Care Walk-In Clinic With An Emergency Department. Dtsch Arztebl Int. 2023;120(29-30):491-498.

Burrell A, Scrimgeour G, Booker M. GP roles in emergency medical services: a systematic mapping review and narrative synthesis. BJGP Open. 2023;7(2).

Gonçalves-Bradley D, Khangura JK, Flodgren G, Perera R, Rowe BH, Shepperd S. Primary care professionals providing non-urgent care in hospital emergency departments. Cochrane Database Syst Rev. 2018;2(2):Cd002097.

Henderson J, Gallagher R, Brown P, Smith D, Tang K. Emergency Department After-Hours Primary Contact Physiotherapy Service Reduces Analgesia And Orthopaedic Referrals While Improving Treatment Times. Aust Health Rev. 2020;44(3):485-492.

Jeyaraman MM, Copstein L, Al-Yousif N, et al. Interventions and strategies involving primary healthcare professionals to manage emergency department overcrowding: a scoping review. BMJ Open. 2021;11(5):e048613.

Jeyaraman MM, Alder RN, Copstein L, et al. Impact of employing primary healthcare professionals in emergency department triage on patient flow outcomes: a systematic review and meta-analysis. BMJ Open. 2022;12(4):e052850.

Magarey AW, Weng J, Looi JCL, Allison S, Bastiampillai T. Systematic Review of Psychiatric Observation Units and Their Impact on Emergency Department Boarding. Prim Care Companion CNS Disord. 2023;25(6).

Noble S, Flattery P, Stonehouse W, Et Al 2046 'Call Before Convey' – Delivering Urgent Care For Patients In The Right Place With The Right Clinician, First Timeemergency Medicine Journal 2023;40:871-872.

Romeo M, Money J, Toloo GS, Lim D. Effectiveness of general practice availability in reducing avoidable utilisation of emergency departments: A rapid review of the literature. 2020.

Scantlebury, A., Adamson, J., Salisbury, C., Brant, H., Anderson, H., Baxter, H. et al. (2022). Do General Practitioners Working In Or Alongside The Emergency Department Improve Clinical Outcomes Or Experience? A Mixed-Methods Study. Bmj Open, 12(9), E063495.

Wackers E, Stadhouders N, Maessen M, et al. Association Between Acute Care Collaborations And Health Care Utilization As Compared To Stand-Alone Facilities In The Netherlands: A Quasi-Experimental Study. Eur J Emerg Med. 2023;30(1):15-20. Doi:10.1097/Mej.0000000000000969

9. Acute Respiratory Infection (ARI) Hubs

Table S9.1: ED waiting time-based metrics (Tier 1)

First author year (ref)	Country & sample size (n=studies)	Study design	Aim	Intervention<br><br>Population	Findings<br><br>Other ED outcomes reported
Burgess, 2021	N = 26 (total)<br><br>Relevant: 5 (Canada, USA)	SR (with MA)	To assess the effects of nurse-initiated interventions on patients' outcomes in ED.	Interventions: ED-based nurse-initiated interventions (e.g., nurse-initiated pathology, nurse-initiated medications, and nurse-initiated intravenous fluid therapy).<br><br>Population: Patients with acute respiratory distress due to asthma or COPD exacerbations.	Waiting-times (n studies = 4)<br>- An asthma management protocol was found to improve time-to-steroids by a mean of 22.8 minutes ($p = 0.01$),<br>- A modified asthma pathway providing corticosteroids was found to reduce the time-to-treatment by 39 minutes ($p < 0.01$).<br>- Introduction of a nurse-initiated steroids scheme at triage was found to reduce time-to-treatment time by a mean reduction of 44 minutes ($p = 0.001$).<br><br>Hospital admissions (n studies = 4; three of them included patients with acute respiratory distress and one study included patients with neonatal jaundice).<br><br>Meta-analysis results (OR, 95%CI)<br>- Nurse-initiated interventions (n studies=3; outcome: hospital admission): **0.51 (0.40, 0.66)**, I^2= 0%

References:

Burgess L, Kynoch K, Theobald K, Keogh S. The effectiveness of nurse-initiated interventions in the Emergency Department: A systematic review. *Australas Emerg Care.* 2021;24(4):248-254.

10. Virtual Wards / Hospital At Home (HAH)

Table S10.1: ED waiting time-based metrics (Tier 1)

First author year (ref)	Country & sample size (n=studies)	Study design	Aim	Intervention<br><br>Population	Findings<br><br>Other ED outcomes reported
Totten, 2019	Total, n=19<br>USA (12), Hong Kong (2), Italy, Japan, Korea, Brazil, Turkey (1)<br><br>Relevant studies, n=1<br>USA (1)	Systematic review	To conduct a systematic review to identify and summarize the available evidence about the effectiveness of telehealth consultations... Telehealth consultations are defined as the use of telehealth to facilitate collaboration between two or more providers, often involving a specialist, or among clinical team members, across time and/or distance	Telehealth: Emergency care specialist consultations, including telepsychiatry<br><br>Mental health	(p.65) psychiatric consults reduced hospital time in the ED (Southard, 2014) ...<br><br>Other ED outcomes: Costs

Table S10.1.1: ED waiting time-based metrics (Tier 2)

First author year (ref)	Country & sample size (n=studies)	Study design	Aim	Methods & Outcomes	Intervention<br><br>Population	Findings<br><br>Other ED outcomes reported
Helberg et al, 2023	USA<br>n=60<br><br>n=40 (Hospital at Home, HAH) and 20 (usual care, UC)	Non-randomised prospective case–controlled	To evaluate the quality and safety of the HAH for heart failure patients	A non-randomised prospective case–controlled of patients enrolled in the HAH versus admission to the hospital (UC).<br><br>Primary outcomes includED-LOS (LOS), adverse events, discharge disposition, patient satisfaction. Secondary outcomes included 30-day readmission rates, 30-day ED usage and ED dwell time.	Patient receives in-person versus telephonic visit once arriving home. Patients were then seen anywhere from three to five times per day by a member of Remote Health Services' HAH team. Initially, at least two of the visits would be conducted by an RN/paramedic in conjunction with a nurse practitioner supervised by a physician for optimisation of their HF regimen, intravenous diuretic therapy and lab work. The majority of patients received two times a day intravenous diuretic therapy during their acute phase and daily or every other day basic metabolic panel depending on underlying renal function. If any issues occurred with medical management, the HF clinic physician was available for further care recommendations…The patient also had access to a Remote Health Services 24/7 call centre and rapid mobile urgent care if needed. After treatment in the acute phase, patients entered a 30-day transitional phase where they were periodically assessed by the HAH team	ED dwell time for those seen in the ED was decreased for those in the HAH even with the addition of the HAH team evaluations (7.1 hours vs 9.3 hours) (non-significant difference).<br><br>Other ED outcomes: patient satisfaction; discharge disposition; readmission rates, ED usage

					Patients presenting to their community providers or ED with symptoms of acute on chronic HF (CHF) requiring admission	

Table S10.2: Ambulance time-based metrics (Tier 1)

First author year (ref)	Country & sample size (n=studies)	Study design	Aim	Intervention<br><br>Population	Findings<br><br>Other ED outcomes reported
Baratloo, 2018	Total, n=26,<br>USA (12), France, UK (3), Germany, Spain (2), Austria, Finland, Hong Kong, Italy (1)<br><br>Relevant studies, n=5<br>USA (2), Germany, Hong Kong, UK (1)	Systematic review	To assess the effects of telemedicine on treatment times and clinical outcomes of acute stroke care	Telemedicine:<br>Pre-hospital telestroke-based systems (Telephone, videoconferencing and teleradiology)<br><br>Stroke patients	Five heterogeneous studies ($p = 0.03$, $I2 = 62\%$, $Tau2 = 35.4$) reported data on OTD duration for telestroke (456 patients) and control (377 patients) groups (Fong et al 2015, Amorim et al 2013, Audebert et al 2006, Chowdhury et al 2012, Uchino et al 2010). Under the random effects model, the overall effect estimate showed that onset to door (OTD) duration was statistically less in the group (MD = −7.19 minutes, 95% CI [−13.89, −0.48], $p = 0.04$), compared to the control group; Figure 3A. Heterogeneity was best resolved by excluding the study by Chowdhury et al (2012) ($p = 0.58$, $I2 = 0\%$, $Tau2 = 0$), while the effect estimate remained significant (MD = −10.4 minutes, 95% CI [−14.79, −6.01], $p < 00001$).Other ED outcomes: Mortality, stroke-based outcomes, LoS hospital
Culmer, 2020	Total, n=13 Germany (4), US (2), Korea (2), UK (1), Belgium (1), Greece (1), Spain (1), international (1)<br><br>Relevant studies, n=3<br>Germany (2), Spain (1)	Systematic review	To examine the clinical importance of telemedicine in patient-provider ambulance-based settings with a focus on multifunctional systems for general prehospital emergency populations.	Telemedicine:<br>Ambulances equipped with telemedicine for general emergency populations (telemedically connected ambulances often capturing audio, video, vital signs, and more if needed or relevant); (e) involved pre-hospital communication between the doctors and paramedics, emergency medical technicians, or patients, which provided a rich communication medium and delivered vitals in<br>real time<br><br>General	Two studies (Langabeer 2016, Cabrera 2002) reported significantly lower response times when using telemedicine, although articles reported travel times differently.<br>Some accounted it as productivity (time in service),(Langabeer 2016, 2017) with results showing telemedicine ambulance responses taking only 39 minutes, compared to a control time of 83 minutes.<br>Another parsed out differences in travel time including five different components (activation, response, on scene, transport, delivery, and recovery) and found a statistically significant reduction of response time in all categories was observed across 100 patients. (Cabrera, 2022) One additional study noted that telemedicine allowed for earlier initiation of treatment (Bergrath, 2013)<br><br>Other ED outcomes: Costs

Totten, 2019	Total, n=13<br>Italy (5), Denmark (2), USA, Germany, Canada, Brazil, Turkey (1)<br><br>Relevant studies, n=10<br>Italy (5), Denmark (2), USA, Canada, Turkey (1)	Systematic review	To conduct a systematic review to identify and summarize the available evidence about the effectiveness of telehealth consultations...<br>Telehealth consultations are defined as the use of telehealth to facilitate collaboration between two or more providers, often involving a specialist, or among clinical team members, across time and/or distance	Telehealth:<br>Emergency medical service/urgent care prehospital telehealth<br><br>Cardiology and cardiovascular (STEMI)	(p.65) and cardiology consultations resulted in faster… treatment (Astarcioglu et al 2015)<br>(p.74) In the studies of prehospital cardiac assessment, nine included a measure of time to treatment (e.g., time to treatment, percent treated within recommended time, total ischemic time), and all of these reported time to treatment was statistically significantly shorter with telehealth (Sanchez-Ross 2011, Brunetti 2014, Martinoni et all 2011, Ortolani 2006 and 2007; Pedersen 2009; Sejersten 2008; Zanini et al 2008; Chan et al 2012)<br><br>Other ED outcomes: Hospital admissions

References:

Baratloo A, Rahimpour L, Abushouk A, Safari S, Lee C, Abdalvand A. Effects of telestroke on thrombolysis times and outcomes: a meta-analysis. *Prehosp Emerg Care.* 2018;22:472-484.

Culmer N, Smith T, Stager C, Meyer H, Quick S, Grimm K. Evaluation of the triple aim of medicine in prehospital telemedicine: A systematic literature review. *J Telemed Telecare.* 2020;26(10):571-580.

Helberg J, Bensimhon D, Katsadouros V, al. e. Heart failure management at home: a non-randomised prospective case–controlled trial (HeMan at Home). *Open Heart.* 2023;10:e002371.

Totten AM, Hansen RN, Wagner J, et al. AHRQ Comparative Effectiveness Reviews. In: *Telehealth for Acute and Chronic Care Consultations.* Rockville (MD): Agency for Healthcare Research and Quality (US); 2019.
